# Ecometabolomics for a Better Understanding of Plant Responses and Acclimation to Abiotic Factors Linked to Global Change

**DOI:** 10.3390/metabo10060239

**Published:** 2020-06-09

**Authors:** Jordi Sardans, Albert Gargallo-Garriga, Otmar Urban, Karel Klem, Tom W.N. Walker, Petr Holub, Ivan A. Janssens, Josep Peñuelas

**Affiliations:** 1 Spain National Research Council (CSIC), Global Ecology Unit CREAF-CSIC-UAB, 08193 Bellaterra, Spain; Albert.gargallo@gmail.com (A.G.-G.); josep.penuelas@uab.cat (J.P.); 2 Centre de Recerca Ecològica i Aplicacions Forestals (CREAF) Institute, 08193 Cerdanyola del vallès, Spain; 3 Global Change Research Institute, Czech Academy of Sciences, Bělidla 986/4a, CZ-60300 Brno, Czech Republic; urban.o@czechglobe.cz (O.U.); klem.k@czechglobe.cz (K.K.); holub.p@czechglobe.cz (P.H.); 4 Department of Environmental Systems Science, Eidgenössische Technische Hochschule (ETH) Zürich, 8092 Zürich, Switzerland; thomas.walker@usys.ethz.ch; 5 Department of Biology, University of Antwerp, 2610 Wilrijk, Belgium; ivan.janssens@uantwerpen.be

**Keywords:** flavonoids, free amino acids, gas chromatography-mass spectrometry (GC-MS), proton nuclear magnetic resonance spectrometry (^1^H-NMR), isoflavonoids, liquid chromatography-mass spectrometry (LC-MS), phenolics, shikimate acid, soluble sugars, terpenes

## Abstract

The number of ecometabolomic studies, which use metabolomic analyses to disentangle organisms’ metabolic responses and acclimation to a changing environment, has grown exponentially in recent years. Here, we review the results and conclusions of ecometabolomic studies on the impacts of four main drivers of global change (increasing frequencies of drought episodes, heat stress, increasing atmospheric carbon dioxide (CO_2_) concentrations and increasing nitrogen (N) loads) on plant metabolism. Ecometabolomic studies of drought effects confirmed findings of previous target studies, in which most changes in metabolism are characterized by increased concentrations of soluble sugars and carbohydrate derivatives and frequently also by elevated concentrations of free amino acids. Secondary metabolites, especially flavonoids and terpenes, also commonly exhibited increased concentrations when drought intensified. Under heat and increasing N loads, soluble amino acids derived from glutamate and glutamine were the most responsive metabolites. Foliar metabolic responses to elevated atmospheric CO_2_ concentrations were dominated by greater production of monosaccharides and associated synthesis of secondary metabolites, such as terpenes, rather than secondary metabolites synthesized along longer sugar pathways involving N-rich precursor molecules, such as those formed from cyclic amino acids and along the shikimate pathway. We suggest that breeding for crop genotypes tolerant to drought and heat stress should be based on their capacity to increase the concentrations of C-rich compounds more than the concentrations of smaller N-rich molecules, such as amino acids. This could facilitate rapid and efficient stress response by reducing protein catabolism without compromising enzymatic capacity or increasing the requirement for re-transcription and de novo biosynthesis of proteins.

## 1. Background

The term “ecometabolomics”, which first appeared in the scientific literature in 2009 [1,2], describes the use of nontarget metabolomics to study the responses, acclimation and adaptation of living organisms to environmental conditions [3,4,5,6]. The advances in nontarget analytical platforms has provided a new tool for ecologists and environmental researchers. Gas chromatography-mass spectrometry (GC-MS), liquid chromatography–mass spectrometry (LC-MS) and nuclear magnetic resonance spectrometry (NMR) are the platforms that are most often used because they offer the best capacity to determine the widest ranging sets of metabolites and thus are the most adequate and used platforms for ecometabolomics. GC-MS has proven to be a robust tool for the study of volatile organic compounds [7,8], but GC-MS analysis of extracts containing other analytes such as organic acids, sugars, amino acids and steroids is more complicated. Many metabolites are nonvolatile and must be derivatized prior to GC-MS analysis [9]. In such cases, thermolabile compounds may be lost. Moreover, it is difficult to elucidate the unknown structures of metabolites by using GC-MS alone. LC-MS is of particular importance for studying a great number of metabolic pathways at once, since this analytical approach has an excellent capacity to separate and determinate semipolar metabolites. This coincides with the fact that the plant metabolism embodies a huge range of semipolar compounds, including many key groups of secondary metabolites, which are thus better separated and detected by LC-MS [10]. Thus, GC-MS is best suited for compound classes appearing mainly in primary metabolism (frequently after derivation, i.e., amino acids, fatty acids and sugars) or for volatile compounds. LC-MS is more adequate for determining the overall biochemical richness of plants including several semipolar groups of secondary metabolites, and thus is the most frequently used platform in ecometabolomics of plants. To gain structural elucidation power, the method of collision-induced dissociation can be used [11], giving rise to the fragment spectrum (MS/MS method) [12]. Fourier-transform ion cyclotron resonance mass spectrometry (FT-ICR-MS) can be used to further increase the number of detectable metabolites. FT-ICR-MS is a very high resolution technique in that masses can be determined with very high accuracy. This is due to the great sensitivity of this method to separate compounds with different mass-to-charge ratios (*m*/*z*) by their different cyclotron frequencies in a fixed magnetic field. This method has been rarely used together with previous chromatographic methods in ecometabolomic studies until recently [13], but the results are promising. ^1^H-NMR has proven to be an appropriate tool for untargeted analyses. It has the advantage that it can be applied to determine polar, semipolar and nonpolar metabolites, and it produces signals that are directly and linearly correlated with compound abundance [14]. However, NMR spectroscopy has intrinsic low sensitivity for low concentrations of metabolites and signal overlapping for complex mixtures. This can at times be problematic for structural elucidation of a metabolite at low concentrations [12]. Thus, although the sensitivity of NMR spectroscopy has increased enormously and improvements continue to emerge steadily, this remains a weak point for NMR compared with MS [12]. MS-based metabolomics provides an excellent approach that can offer a combined sensitivity and selectivity platform for metabolomics research. Moreover, different MS approaches, such as different ionization techniques and mass analyzer technologies, can be used in order to increase the number of metabolites that can be detected, making this the most frequent analytical technique used in metabolomics studies [12].

While the early use of ecometabolomics has been reviewed previously [3,15], there has been a recent explosion in the number of ecometabolomics studies undertaken in specific environmental fields, such as abiotic stresses in crop plants [16,17], plant responses to pathogens and toxic chemicals [18,19,20], plant resistance to salt stress [21], plant responses to cold and heat stress [22], plant tolerance to metals and metalloids [23], N eutrophication [24,25] and elevated atmospheric CO_2_ [26,27]. There is thus a need to review both what has been learned from these studies and to explore the potential for applying ecometabolomics in future research. Thus, ecometabolomics can provide important knowledge regarding the acclimation responses to stress at the level of primary metabolism shifts and regulation of secondary metabolites related to antioxidants and defense against stress [1,3,5,17,19]. Furthermore, ecometabolomics should provide clues for breeding and selection in evolutionary and epigenetic processes [1,3,5,17,19].

Here, we aimed to summarize the usage of ecometabolomics in understanding plant responses and adaptation to four key global change factors, namely drought, climate warming, atmospheric CO_2_ concentrations and N eutrophication. These factors were chosen because they change at rates that outpace the evolution of phenotypic plasticity in organisms. In doing so, we also identify knowledge gaps and areas for future research.

## 2. Drought

Arid and semiarid lands currently occupy about 41% of the global land surface [28], and socioeconomic and climatic modeling predicts that the arid land surface area may increase by 11–23% by the end of this century, reaching between 50–56% of the total land surface area under more probable RCP4.5 and RCP8.5 scenarios [29]. We must take into account that RCP4.5 scenario peak of CO_2_ emissions will occur around 2040, then decline, whereas the emissions continue to rise throughout the full 21st century in the RCP8.5 scenario. As the most used scenarios in projection modeling, the RCP4.5 projection represents the most “optimistic but possible” scenario, while the RCP8.5 represents the most pessimistic scenario. Plants in affected regions must respond rapidly to higher levels of drought stress if they are to acclimate and survive. Thus, understanding plant species’ metabolic shifts in response to drought will allow prediction and mitigation of ecological change in wild and crop plant communities under scenarios of increased drought. In addition, changes in plant metabolome may affect community composition and ecosystem trophic webs through shifts in the nutritional value of plant tissues or through the accumulation of secondary protective compounds that may play a role as attractants/repellents, triggered by acclimation mechanisms to environmental change [2,5,21]. Gaining a deeper understanding of metabolic adaptive strategies to short-term drought effects may thus be useful in the selection of novel drought-resistant genotypes of crop plant species.

**Figure 1:**
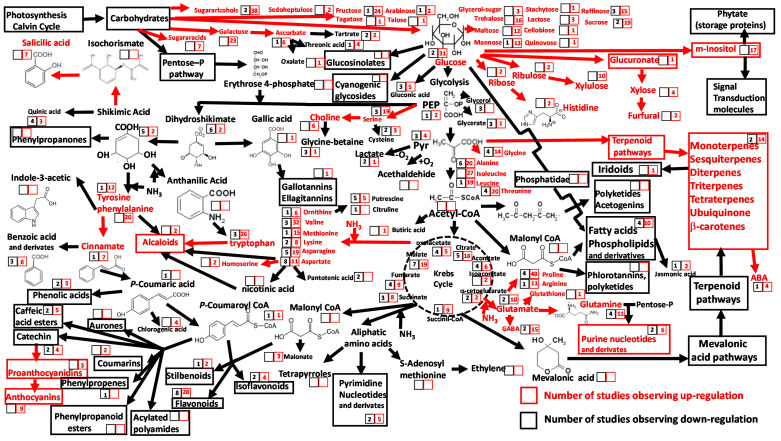
Shifts in principal plant metabolites and metabolic pathways in response to drought conditions reported in 81 ecometabolomic studies. The numbers within the squares are the numbers of reports describing changes in photosynthetic tissue metabolite concentration and/or pathway activity under drought stress. Red numbers within red squares indicate significant increases; black numbers within black squares indicate significant decreases. Red arrows indicate upregulation of the metabolic pathway; bold black arrows indicate no changes.

A growing number of studies have used nontarget metabolomics techniques to analyze metabolic shifts in plant photosynthetic tissues under drier conditions (Figure 1, Table S1). There are clear increases and decreases in specific metabolites and metabolite groups in response to drying. Specifically, most studies report increased foliar concentrations of some monosaccharides and disaccharides, including their direct derivatives, and many report higher concentrations of sugar alcohols (both acyclic and cyclic) and sugar acid derivatives, along with oligosaccharides such as raffinose and stachyose, under higher drought intensity [30,31,32,33]. The pathways from glucose to xylulose, xylose, inositol and several polyols and sugar alcohols are activated in several plant species under drought stress. Polyols, such as sorbitol and mannitol, are osmoprotectants [34,35], and some, including mannitol, act as enzyme protectants (thiol-regulated enzymes) against inactivation by free radicals, such as hydroxyl radicals. This is important because hydroxyl radicals increase in abundance under drought-induced oxidative stress [36]. The role of sugars and sugar alcohols as osmolytes has been widely reported in plant antistress acclimation [37,38]. High concentrations of glucose and other soluble sugars could reflect a need for sugar reserves to supply energy during stress events and minimize negative effects of stress [28]. Accumulations of sugars and alcohol sugars that are accompanied by increases in concentrations of organic acids in Krebs cycle metabolism, which have been observed in some studies, do not appear to be consistent under drought stress [37].

A second group of plant metabolites frequently observed at greater concentrations under drought stress comprises a diverse array of amino acids, particularly those derived from pyruvate, phosphoenolpyruvate, oxaloacetate, shikimic acid and α-ketoglutaric acid (Figure 1). These include tryptophan, isoleucine and proline [39,40,41,42]. Although foliar concentrations of other amino acids, such as aspartate [42] and alanine [43], have been shown to decrease under drought stress, total amino acid concentrations generally increase in response to drought conditions. We found that proline was the most frequently observed upregulated amino acid under drought stress [35,44,45,46], probably in response to reduced carbon (C) and nitrogen (N) storage that occurs during drought stress [47]. Increases in choline and serine concentrations have also been reported [48,49] (Figure 1, Table S1). The large number of studies showing rises in proline concentrations under drought are consistent with the demonstrated role of proline as osmolyte and antioxidant against multiple stresses [50]. There was limited evidence, from only three studies (Table S1) that the osmoprotectant glycine-betaine and its derivatives occurred at higher concentrations under drought conditions. However, one study showed that drought increased concentrations of glycine-betaine, supporting its role in drought stress [46,51]. Choline, a precursor to glycine-betaine, has also been reported to increase in concentration under drought (Figure 1, Table S1). In contrast, aspartate is unlikely to play a role as osmoprotectant during drought [2,51,52], with eight studies reporting increases and seven studies reporting decreases. Finally, there is strong evidence for the production of nonaliphatic amino acids involved in the shikimic acid pathway, given that foliar tissue concentrations of these amino acids consistently increase under drought conditions (Figure 1, Table S1). These aromatic amino acids and specially tryptophan are involved in drought antistress mechanisms [53,54].

A key finding from ecometabolomic studies is the observation that drought induces an increase in foliar concentrations of proline, arginine and glutamine in the glutamate pathway (Figure 1, Table S1). Consistent with its role as a precursor of γ-aminobutyric acid (GABA), higher concentrations of glutamate typically accompany elevated concentrations of GABA in drought-treated plants (Figure 1). This result is consistent with the role of GABA in the control of stomatal closure, especially under stress [55,56,57]. Although metabolomic and other analytical methods have yielded data consistently showing higher concentrations of amino acids from the pyruvate and oxaloacetate pathways, no clear patterns of organic acid concentrations in the Krebs cycle are apparent [57]. The results also indicate a trend towards higher ABA concentrations (Figure 1). ABA triggers a cascade of effects, terminating in stomatal closure [35], and is involved in the upregulation of genes involved in the formation of proline from glutamate [58].

Fatty acids concentration increases have been also associated to drought acclimation [59]. Increased production of osmoprotectants occurred as an immediate response, but lipid metabolism also shifts as the drought continues since the plant needs to adapt its membranes as well [59]. In fact, ten studies have observed increases in fatty acids concentration, and only four studies have observed decreases (Figure 1). The timing of drought and sampling may be behind these contradictory results.

Three clear effects of drought stress on secondary metabolites have been revealed by ecometabolomic studies. First, there is stronger evidence for increases, rather than decreases, in concentrations of terpenes and flavonoid groups such as anthocyanins under drought stress (Figure 1). Specifically, four out of five studies have shown increases in concentrations of terpenes. These results were to be expected, because terpenes act as antioxidant molecules [34]. Although some recent reviews have observed both increases and decreases in foliar concentrations of terpenes and their associated derivatives under contrasting levels of drought stress [60], most studies have shown higher terpene concentrations under drought stress (Figure 1, Table S1). Second, a recent meta-analysis of 1475 studies [61] showed that, among secondary metabolites, only concentrations of flavonoids tended to increase under drought. This finding is consistent with our evaluation of 28 out of 30 ecometabolomics studies showing rises of flavonoid concentrations in photosynthetic tissues under drought conditions, with only two studies reporting decreased concentrations (Figure 1, Table S1). Third, ecometabolomics studies generally reported an upregulation of salicylic acid (seven studies) in response to drought. Salicylic acid is a product of the shikimic acid pathway and, along with other nonaliphatic amino acids, has been shown to alleviate drought-mediated damage [62]. Jasmonic acid concentrations increased in four studies and decreased in only one study in response to drought (Figure 1, Table S1).

Some of these contradictory results can be explained by the differences between short-term and mid- to long-term responses to drought acclimation. Jasmonic acid, which stimulates glutathione metabolism, may be triggered by ABA accumulations [63,64]. The observed discrepancies in drought responses, such as those reported for jasmonic acid, could be due to the fact that metabolic changes induced by moderate stress may promote the biosynthesis of jasmonic acid, whereas severe stress may imply degradation of flavonoids during scavenging of free radicals [65].

Finally, of the reviewed studies using an NMR platform approach to study drought effects of plant metabolism, five studies occurred prior to 2014 and four occurred after. Meanwhile, of the studies using chromatography separation followed by MS detection, 22 were completed prior to 2014 and 49 occurred thereafter. This clearly shows the increasing preference for chromatography and MS platforms over NMR platforms.

In conclusion, ecometabolomic studies have collectively shown that plant responses to drought stress involve a system based on increases in concentrations of osmoprotectants and oxidative protectants such as amino acids and N-rich molecules that are coupled to protein catabolism and/or reduction in protein biosynthesis [37], with a rise in sugars and alcohol sugars also being noted. At the same time, increases in terpene, flavonoid and anthocyanin concentrations, including their derivatives, represent larger C-rich secondary compounds that are not coupled to protein catabolism. Thus, the breeding of genotypes with a greater capacity for drought tolerance should focus on those in which concentrations of C-rich compounds increase to a greater extent than those of smaller N-rich molecules. Such a strategy should facilitate a rapid response to drought stress without compromising enzymatic capacity or increasing the requirement for re-transcription and de novo biosynthesis of proteins.

## 3. Heat Stress

**Figure 2:**
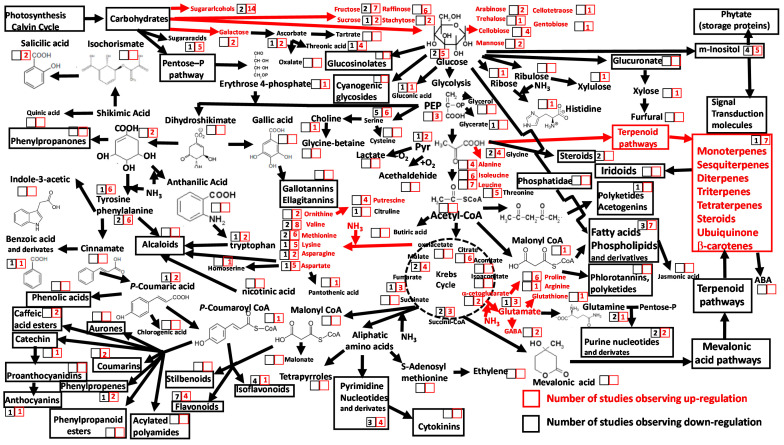
Shifts in principal plant metabolites and metabolic pathways in response to heat stress reported in 22 ecometabolomic studies. The numbers within the squares are the numbers of reports describing changes in photosynthetic tissue metabolite concentration and/or pathway activity under heat stress. Red numbers within red squares indicate significant increases; black numbers within black squares indicate significant decreases. Red arrows indicate upregulation of the metabolic pathway.

As heat stress becomes an increasing challenge under projected global warming, with a greater frequency and intensity of heat waves [66], research on methods to improve crop resistance to heat events is of paramount importance but remains a scientific challenge [67,68]. Heat stress in plants stimulates the production of reactive oxygen species (ROS) that damage a wide array of cellular components through disrupted membrane stability or reduced energy metabolism [69,70]. As a result, ROS trigger oxidative stress responses [71,72,73]. Similarly to drought, heat stress has been shown to increase concentrations of soluble carbohydrates in photosynthetic tissue, such as sugar metabolites and associated derivates [73,74] and, in particular, sugar alcohols, fructose and raffinose (Figure 2, Table S2). The findings of these metabolomic analyses are consistent with earlier studies of plant physiological responses to heat stress, which found a reduction in photosynthesis and an increase in respiration [75,76], mediated through the mobilization of nonstructural polysaccharides. This mobilization drives increases in cellular concentrations of soluble carbohydrates, available as C-skeletons for catabolic and anabolic reactions, improving osmotic and antioxidant protection [75,76,77].

Ecometabolomic studies have also revealed high concentrations of amino acids in foliar tissue in response to heat stress, especially those synthesized in the pyruvate and α-ketoglutarate-glutamate pathway, such as proline, GABA, glutathione, alanine, isoleucine and leucine (Figure 2, Table S2). Heat stress effects on proline concentrations in plants have been widely studied [78,79] and have shown the importance of proline in osmotic adjustment [80], protection of protein structures [81] and scavenging of free oxidative radicals [82]. In *Arabidopsis*, glutathione controls expression of heat shock protein genes [83], and, in wheat and maize, it acts as an electron donor for free radical scavenging [84]. We found that few ecometabolomics studies have reported a rise in glutathione concentration, but this could be because all glutamate-related metabolites are detected at higher concentrations under heat stress. Of these, GABA has also been shown to accumulate in *Arabidopsis* roots under heat stress [85]. In fact, studies applying drought and heat at the same time have observed a rise in sugars and free amino acids, reinforcing the useful role of these groups of primary metabolites for short-term adaptation to these stresses [59].

Ecometabolomic analyses have revealed a lack of consistency in changes in flavonoid foliar tissue concentrations (total or as individual compounds) in response to heat stress (Figure 2, Table S2). For example, several studies have shown that changes in phenolic compounds and derivatives, such as anthocyanins, under increasing temperatures depend rather on the magnitude of temperature change, the organ in question and other conditions such as light or water availability than on the particular temperature value [86,87,88,89]. Rising terpene concentrations in leaves under high temperatures [90] were confirmed by ecometabolomic studies, indicating that the biosynthesis of isopentyl diphosphate from pyruvate and glyceraldehide-3-P relates to heat stress avoidance. This result is consistent with the role of isopentyl diphosphate in the synthesis of terpenoids and carotenoids in antioxidant responses to oxidative stress [91,92].

Metabolomic studies provided evidence for shifts in the plant metabolome under heat stress. Increased concentrations of soluble sugars and amino acids are generalized heat response mechanisms across several plant species. Accumulation of primary metabolites, such as carbohydrates, in response to heat stress increases the stability of proteins and the cell membrane bilayer structure [93]. Thus, strategies that develop and select for phenotypes with efficient protective mechanisms against heat stress, such as those that trigger increases in soluble carbohydrates, should be encouraged in contrast with those that trigger increases in free amino acids. Carbohydrates can be obtained from nonfunctional reserves, whereas free amino acids come from protein catabolism or from decreased de novo biosynthesis of proteins. An increase in the capacity of a fast synthesis of fatty acids that protect from cell damage is also suggested as a mechanism against heat stress.

## 4. Elevated Atmospheric CO_2_ Concentrations

**Figure 3:**
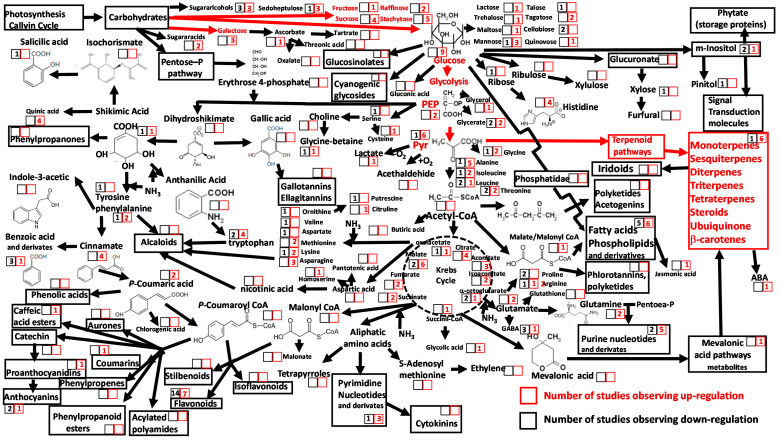
Shifts in principal plant metabolites and metabolic pathways in response to increased atmospheric CO_2_ concentrations reported in 17 ecometabolomic studies. The numbers within the squares are the numbers of reports describing changes in photosynthetic tissue metabolite concentration and/or pathway activity under elevated atmospheric CO_2_ concentrations. Red numbers within red squares indicate significant increases; black numbers within black squares indicate significant decreases. Red arrows indicate upregulation of the metabolic pathway.

Atmospheric CO_2_ concentrations are rising continuously [94], and several impacts on plant functioning, such as increased water and nutrient use efficiency [95,96,97], a positive effect on photosynthesis rates [98,99] and changes in chemical foliar composition [100,101,102,103], have been associated to this rise. Ecometabolomic studies show that metabolites that increase most markedly in plant leaves under elevated atmospheric CO_2_ are terpenes and soluble sugars, particularly the galactose, glucose, and fructose monosaccharides (Figure 3, Table S3). This indicates that increases in newly photosynthesized carbon triggers immediate accumulations of photosynthetic products (glucose and directly associated molecules). Previous studies have suggested that elevated atmospheric CO_2_ concentrations lead to increases in secondary metabolites from the shikimic acid pathway, such as phenolics [74,75,76,77,78,79,100,104,105,106,107,108]. However, with the exception of quinic and cinnamic acids, which were consistently observed to increase with CO_2_ concentrations (Figure 3), we found contrasting observations among ecometabolomic studies (Figure 3, Table S3). Specifically, despite earlier evidence for positive links between rises in atmospheric CO_2_ concentrations and C-rich secondary compounds [100,107,109], data from ecometabolomic studies revealed that this relationship varies among compounds and depends on interactions with other environmental variables. Moreover, these recent metabolomics studies all reported increases in concentrations of salicylates and phenolic acids [110], anthocyanins, and flavonoids [111,112] under doubled CO_2_ concentration, at least twice those observed under current levels of ambient [CO_2_] [113,114]. Thus, plant responses to high concentrations of atmospheric CO_2_ are specific between secondary metabolite families and depend on associated environmental conditions.

Rises in concentrations of foliar terpenes under increased levels of atmospheric CO_2_ have been observed using targeted analyses [100,101,106,115]. This rise can, however, be also a partial indirect response to warming coupled to atmospheric CO_2_ concentration enhancement [116,117]. Terpene production is dependent on sugar metabolism. As such, increases in terpene concentrations under elevated atmospheric CO_2_ are likely to be a result of associated increases in soluble monosaccharides (Figure 3). Although there have been limited reports of decreases in terpene concentrations in plants under high CO_2_ atmospheric concentrations [118], the consensus from this review of ecometabolomic studies is that plant responses to elevated CO_2_ are likely mediated by rises in monosaccharide production and by associated secondary metabolite groups, such as terpenes, that are directly synthesized from monosaccharides. In contrast, other secondary compounds synthesized by longer pathways from other sugars and those involving N-rich molecule precursors, such as cyclic amino acids and those in the shikimate pathway, should be not upregulated under elevated atmospheric CO_2_ concentrations (Figure 2, Table S3). Decreases in N concentrations and increases in C:N ratios are common in plant organs under elevated atmospheric CO_2_ [100,107]; however, no clear patterns have been reported for key small organic N-rich molecules, such as amino acids and nucleosides, with almost equal numbers of studies reporting positive and negative responses (Figure 3, Table S3). Elevated atmospheric CO_2_ concentrations counteract the effect of drought and heat in producing high concentrations of sugars and free amino acids in leaves of *Arabidopsis* [72].

## 5. Nitrogen Eutrophication

**Figure 4:**
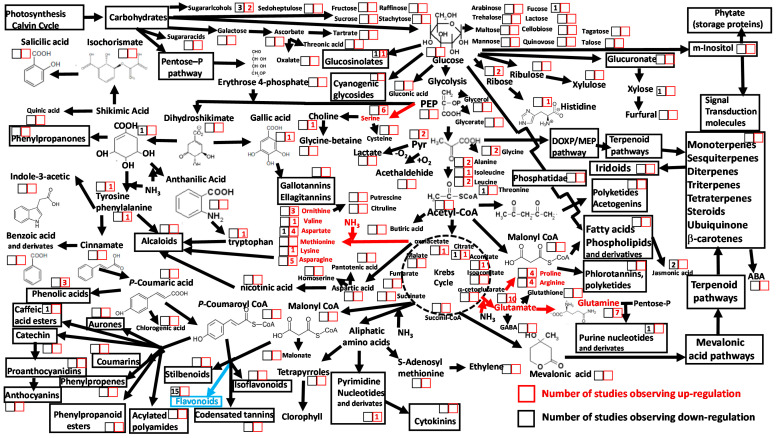
Shifts in principal plant metabolites and metabolic pathways in response to increased N loads reported in eight ecometabolomic studies. The numbers within the squares are the numbers of reports describing changes in photosynthetic tissue metabolite concentration and/or pathway activity under higher N loads. Red numbers within red squares indicate significant increases; black numbers within black squares indicate significant decreases. Red arrows indicate upregulation of the metabolic pathway. Blue arrows indicate downregulation of the metabolic pathway.

Global data clearly show an exponential rise in human-driven N emissions [103], which in natural vegetation has mediated a diversity of effects on productivity, species composition, nutrient cycling and soil processes [119,120,121]. In agriculture, long-term N fertilization in crops has enhanced yields [122,123] and increased total protein and amino acid concentrations in food crops [124]. Targeted analyses based on colorimetric and/or HPLC-UV detection have shown that increases in soil N availability, such as those caused by N deposition and the application of N fertilizer, have strong positive impacts on amino acid concentrations in crops [125,126,127,128,129,130,131,132,133]. Ecometabolomic analyses allow quantification of changes in specific groups of amino acids and their precursor metabolites in response to elevated N loads. Current metabolomics analyses clearly identify the links between elevated N loads and increases in foliar concentrations of free dissolved amino acids, as well as decreases in some groups of C-rich metabolites, such as flavonoids (Figure 4, Table S4). This observed decrease in flavonoid biosynthesis could be associated to the reduced activity of phenylalanine ammonia lyase, which under low N releases N from phenylalanine by the production of secondary metabolites [134].

Ecometabolomic studies reporting higher glutamate and glutamine concentrations under greater N supply (Figure 4, Table S4) are consistent with previous targeted studies. Glutamine is an N transporter and precursor of other amino acids, nucleotides and chlorophyll [135,136]. Target analyses have also shown that free glutamate and glutamine, including associated amino acids such as arginine, are used by plants as N storage compounds under elevated N deposition conditions [125,131,137]. N fertilization and N deposition above ambient levels has led to increases in arginine concentrations in needles of conifer trees [138,139], allowing subsequent storage of arginine-rich proteins [139]. More generally, plant species from N poor habitats accumulate ammonium NH_4_^+^ in arginine as NH_4_^+^ availability increases, while plants from N-rich environments tend to accumulate NH_4_^+^ in asparagine [140]. Under high N loads, increased plant N uptake may yield toxic accumulation of NH_4_^+^ [141]. As such, it is likely that the transformation of NH_4_^+^ into amino acids protects plants against excessive accumulation of free NH_4_^+^ [127]. Neutralization of NH_4_^+^ in amino acids may be more efficient in the presence of metabolites common to primary metabolic pathways, such as glycolysis and/or the Krebs cycle. Moreover, analysis of ecometabolomic studies further allows us to suggest that amino acids formed during ammonification of pyruvate (such as glycine, alanine, isoleucine and leucine), α-ketoglutarate (glutamate, proline, arginine and glutamine) and oxoglutarate (ornithine, valine, aspartate, methionine, lysine and asparagine) occur at greater concentrations under higher levels of N load than those derived from longer and more complex metabolic pathways, such as tyrosine, phenylalanine and tryptophan.

## 6. Concluding Remarks

Ecometabolomic studies examining the impacts of drought, heat, elevated atmospheric CO_2_ concentrations and N eutrophication on plant foliar metabolism have already led to greater understanding regarding plant responses to global change. Under drought conditions, metabolic changes appear to be dominated by increases in soluble sugars and derivatives and, following this, a rise in total free amino acid concentrations. Similar increases are frequently observed in plants under heat stress. Given that changes in sugar concentrations were consistent across species, we suggested the selection of potential drought- and heat-tolerant crop genotypes in which C-rich compounds respond more efficiently than N-rich molecules to facilitate rapid response to drought stress, without a reduction in of enzymatic capacity and the subsequent need for re-transcription and de novo biosynthesis of proteins after the stress. We found that flavonoids, anthocyanins and terpenes are secondary metabolites that are most commonly increased under drought conditions. In contrast, both climate warming and increasing N loads are expected to lead to increased foliar concentrations of soluble amino acids derived from glutamate and glutamine. Under drought and heat stress, several studies have found higher concentrations of proline, which has roles as an osmolyte and as a scavenger of free oxidative radicals. Elevated atmospheric CO_2_ concentration generally increases foliar free sugar and terpene concentrations but does not have clear effects on other secondary compound concentrations. We suggest that this response may be mediated by rises in monosaccharide production and associated increases in secondary metabolites, such as terpenes, rather than those that are synthesized along longer pathways from sugars involving N-rich precursor molecules, such as those formed from cyclic amino acids and in the shikimate pathway.

The review of current bibliography shows some key points that should be improved and deeply studied to obtain more consistent information from ecometabolomic studies. First, most current metabolomics studies are not conducted in real wild conditions, and there is a great disproportion of studies between noncrop versus crop species. Most studies, i.e., 67.7%, have been conducted in crop, forage or prototype plants (e.g., *Arabidopsis thaliana*, *Brachypodium distachyon* or *Lotus japonica*). More studies should be conducted on wild plants and, if possible, sample in natural field conditions and not in greenhouses or common gardens. Second, another interesting point can be derived from the fact that the purpose of ecometabolomic studies is to provide a picture of the whole metabolism and its shifts (at least those of the most important metabolic pathways), making it necessary to have easy-to-use and free software allowing the statistical and metabolic pathway analyses. A third piece of potential advice relates to the case of heat stress, where most studies work at temperatures such as 20 °C or above, using controls that are not realistic and applicable to the current warming scenarios. There is a lack of studies that work in field conditions under chronic moderate warming conditions in a realistic scenario, and there is also a lack of studies considering the combination of field conditions with two or more global change drivers, such as warming and drought, or warming and N and/or CO_2_ rises, in a realistic scenario.

We can also highlight an increasing trend in the use of chromatography coupled to MS detection and a decreasing trend in the use of NMR techniques. In this regard, we have observed that 13.2% of the studies considered in this review that were completed by the end of 2014 had used NMR analyses, whereas this proportion fell to 4.7% after 2014. In contrast, chromatography separation coupled to MS detectors was used in 86.8% of the considered manuscripts completed before 2014 and in 95.3% of the considered manuscripts completed after 2014 (Tables S1–S4).

In summary, ecometabolomics provides a powerful tool not only for understanding responses of plants to global change, but also for identifying potential traits for future crop selection. Furthermore, potential plant community species composition shifts can be projected by comparing the capacity of different plant species to acclimate to environmental shifts. Ecometabolomic studies are thus making substantial steps forward to become an essential tool for ecologists and environmentalists.

## 7. Material and Methods

### Data Collection

We searched the ISI Web of Science using combinations of the following keywords: “ecometabolomic”, “metabolomics”, “metabonomic”, “metabolism”, “plant”, “foliar”, “leaf”, “needle”, “LC-MS”, “GC-MS”, “NMR”, “sugar”, “amino acid”, “lipid”, fatty acid”, “food”, “dry”, “drier”, “drought”, “aridity”, “warm”, “warming”, “heat”, “temperature”, “concentration”, “contents”, “CO_2_”, “metabolite”, “omic”, “N”, “deposition”, “fertilization”, “crop”, “yield”, “terpene”, “alkaloid”, “phenolics”, “flavonoids”, “isoflavonoid”, “free”, “anthocyanins” and “anthocyanidins”. We obtained 367 manuscripts. Thereafter, we conducted a first selection draft where we selected those studies (130) that clearly stablished a treatment of water availability, temperature, atmospheric CO_2_ concentration or N availability and provided data for control and treated plants, so they allowed adequate statistical analyses of the changes in metabolite concentrations and/or metabolic pathway up- and down-regulation (see Tables S1–S4).

## Supplementary Materials

The following are available online at https://www.mdpi.com/2218-1989/10/6/239/s1, Table S1: Drought impacts on higher plant metabolism; Table S2: Heat (warming) impacts on higher plant metabolism; Table S3: CO2; Table S4: N-deposition (loads).

## References

[1] Penuelas J., Sardans J. (2009). Ecological metabolomics. Chem. Ecol..

[2] Kucina V., Ekstron C.T., Anderson S.B., Nielsen J.K., Olsen C.E., Bak S. (2009). Identification of defense compounds in Barberea vulgaris against the Herbivore Phyllotreta nemorum by an ecometabolomic approach. Plant Physiol..

[3] Sardans J., Peñuelas J., Rivas-Ubach A. (2011). Ecological metabolomics as a proxy for organisms, populations, and species lifestyle: Current development and future challenges. Chemoecology.

[4] Rivas-Ubach A., Pérez-Trujillo M., Sardans J., Gargallo-Garriga A., Parella T., Penuelas J. (2013). Ecometabolomics: Optimized NMR-based method. Methods Ecol. Evol..

[5] Rivas-Ubach A., Peñuelas J., Hódar J.A., Oravec M., Tolic L.P., Urban O., Sardans J. (2018). We Are What We Eat: A Stoichiometric and Ecometabolomic Study of Caterpillars Feeding on Two Pine Subspecies of Pinus sylvestris. Int. J. Mol. Sci..

[6] Allevato D.M., Kiyota E., Mazzafera P., Nixon K.C. (2019). Ecometabolomic Analysis of Wild Populations of Pilocarpus pennatifolius (Rutaceae) Using Unimodal Analyses. Front. Plant Sci..

[7] Ozawa R., Shiojiri K., Sabelis M.W., Takabayashi J. (2008). Maize plants sprayed with either jasmonic acid or its precursor, methyl linolenate, attract armyworm parasitoids, but the composition of attractants differs. Èntomol. Exp. Appl..

[8] Llusià J., Penuelas J., Sardans J., Owen S.M. (2010). Niinemets, Ülo Measurement of volatile terpene emissions in 70 dominant vascular plant species in Hawaii: Aliens emit more than natives. Glob. Ecol. Biogeogr..

[9] Gullberg J., Jonsson P., Nordstrom A., Sjöström M., Moritz T. (2004). Design of experiments: An efficient strategy to identify factors influencing extraction and derivatization of Arabidopsis thaliana samples in metabolomic studies with gas chromatography/mass spectrometry. Anal. Biochem..

[10] Allwood J.W., Goodacre R. (2010). An introduction to liquid chromatographya mass spectrometry instrumentation applied in plant metabolomic analyses. Phytochem. Anal..

[11] Jennings K.R. (2000). The changing impact of the collision-induced decomposition of ions on mass spectrometry. Int. J. Mass Spectrom..

[12] Emwas A.-H., Roy R., McKay R., Tenori L., Saccenti E., Gowda G.A.N., Raftery D., AlAhmari F., Jaremko Ł., Jaremko M. (2019). NMR Spectroscopy for Metabolomics Research. Metabolites.

[13] Sousa Silva M., Cordeiro C., Roessner U., Figuereido A. (2019). Editorial: Metabolomics in crop research-current and emerging methodologies. Front. Plant Sci..

[14] Lewis I.A., Schommer S.C., Hodis B., Robb K.A., Tonelli M., Westler W.M., Sussman M.R., Markley J.L. (2007). Method for Determining Molar Concentrations of Metabolites in Complex Solutions from Two-Dimensional1H−13C NMR Spectra. Anal. Chem..

[15] Viant M.R., Bearden D.W., Bundy J.G., Burton I.W., Collette T.W., Ekman E.R., Ezernieks V., Karakach T., Lin C.-Y., Rochfort S. (2009). International NMR-Based Environmental Metabolomics Intercomparison Exercise. Environ. Sci. Technol..

[16] Khakimov B., Bak S., Engelsen S.B. (2014). High-throughput cereal metabolomics: Current analytical technologies, challenges, and perspectives. J. Cereal Sci..

[17] Zhuang J., Zhang J., Hou X., Wang F., Xiong F. (2014). Transcriptomic, Proteomic, Metabolomic and Functional Genomic Approaches for the Study of Abiotic Stress in Vegetable Crops. Crit. Rev. Plant Sci..

[18] Aliferis K.A., Chrysayi-Takousbalides M. (2011). Metabolomics in pesticide research and development: Review and future perspectives. Metabolomics.

[19] Kumar M., Kuzhiumparambil U., Pernice M., Jiang Z., Ralph P. (2016). Metabolomics: An emerging frontier of systems biology in marine macrophytes. Algal Res..

[20] Tugizimana F., Mhlongo M.I., Piater L.A., Dubery I.A. (2018). Metabolomics in Plant Priming Research: The Way Forward?. Int. J. Mol. Sci..

[21] Kumari A., Das P., Parida A.K., Agarwal P.K. (2015). Proteomics, metabolomics, and ionomics perspectives of salinity tolerance in halophytes. Front. Plant Sci..

[22] Gandhi S., Khushu S., Tripathi R.P. (2013). Current metabolomic methodologies and their application to thermal stress. Curr. Metabol..

[23] Jones O.A., Dias D.A., Callahan D., Kouremenos K.A., Beale D.J., Roessner U. (2015). The use of metabolomics in the study of metals in biological systems. Metallomics.

[24] Paudel J.R., Amirizian A., Krosse S., Giddings J., Ismail S.A.A., Xia J., Gloer J.B., van Dam N.M., Bede J.C. (2016). Effect of atmopspheric carbon dioxide levels and nitrate fertilization on glucosinolate biosynthesis in mechanically damaged Arabidopsis plants. BMC Plant Biol..

[25] Hu Y., Peuke A.D., Zhao X., Yan J., Li C. (2019). Effects of simulated atmospheric nitrogen deposition on foliar chemistry and physiology of hybrid poplar seedlings. Plant Physiol. Biochem..

[26] De Souza A.P., Cocuron J.-C., Garcia A.C., Alonso A.P., Buckeridge M.S. (2015). Changes in whole-plant metabolism during the grain-filling stage in sorghum grown under elevated CO2 and drought. Plant Physiol..

[27] Austen N., Walker H.J., Lake J.A., Phoenix G.K., Cameron D.D. (2019). The Regulation of Plant Secondary Metabolism in Response to Abiotic Stress: Interactions Between Heat Shock and Elevated CO2. Front. Plant Sci..

[28] Feng S., Fu Q. (2013). Expansion of global drylands under a warming climate. Atmos. Chem. Phys. Discuss..

[29] Huang J., Yu H., Guan X., Wang G., Guo R. (2015). Accelerated dryland expansion under climate change. Nat. Clim. Chang..

[30] Rivas-Ubach A., Sardans J., Pérez-Trujillo M., Estiarte M., Peñuelas J. (2012). Strong relationship between elemental sotichiometry and metabolome in plants. Proc. Nat. Acad. Sci. USA.

[31] Rivas-Ubach A., Sardans J., Gargallo-Garriga A., Parella T., Perez-Trujillo M., Estiarte M., Penuelas J. (2014). Drought stress enhances folivory by shifting foliar metabolomes in Quercus ilex trees. New Phytol..

[32] Ullah N., Yüce M., Gökçe Z.N.O., Budak H. (2017). Comparative metabolite profiling of drought stress in roots and leaves of seven Triticeae species. BMC Genom..

[33] Shahbazy M., Moradi P., Ertaylan G., Zahraei A., Kompany-Zareh M. (2020). FTICR mass spectrometry-based multivariate analysis to explore distinctive metabolites and metabolic pathways: A comprehensive bioanalytical strategy toward time-course metabolic profiling of Thymus vulgaris plants responding to drought stress. Plant Sci..

[34] Bianco R.L., Rieger M., Sung S.-J.S. (2000). Effect of drought on sorbitol and sucrose metabolism in sinks and sources of peach. Physiol. Plant..

[35] Chaves M.M., Marôco J., Pereira J., Chaves M.M. (2003). Understanding plant responses to drought from genes to the whole plant. Funct. Plant Boil..

[36] Shen B., Jensen R.G., Bohnert H.J. (1997). Mannitol Protects against Oxidation by Hydroxyl Radicals. Plant Physiol..

[37] Llanes A., Andrade A., Alemano S., Luna V. (2018). Metabolomic Approach to Understand Plant Adaptations to Water and Salt Stress. Plant Metab. Regul. Under Env. Stress.

[38] Keunen E., Peshev D., Vangronsveld J., Ende W.V.D., Cuypers A. (2013). Plant sugars are crucial players in the oxidative challenge during abiotic stress: Extending the traditional concept. Plant Cell Environ..

[39] Alvarez S., Marsh E.L., Schroeder S.G., Schachtman D. (2008). Metabolomic and proteomic changes in the xylem sap of maize under drought. Plant Cell Environ..

[40] Barchet G.L., Dauwe R., Guy R.D., Schroeder W., Soolanayakanahally R.Y., Campbell M.M., Mansfield S.D. (2013). Investigating the drought-stress response of hybrid poplar genotypes by metabolite profiling. Tree Physiol..

[41] Gargallo-Garriga A., Preece C., Sardans J., Oravec M., Urban O., Penuelas J. (2018). Root exudate metabolomes change under drought and show limited capacity for recovery. Sci. Rep..

[42] Nakabayashi R., Mori T., Saito K. (2014). Alteration of flavonoid accumulation under drought stress in Arabidopsis thaliana. Plant Signal. Behav..

[43] Pavli O.I., Vlachos C.E., Kalloniati C., Flemetakis E., Skaracis G.N. (2013). Metabolite profiling reveals the effect of drought on sorghum (Sorghum bicolor L. Moench) metabolism. Plant Omics J..

[44] Hsiao T.C. (1973). Plant responses to water stress. Ann. Rev. Plant Physiol..

[45] Fathi A., Tari D.B. (2016). Effect of Drought Stress, and its Mechanism in Plants. Int. J. Life Sci..

[46] Ahanger M.A., Gul F., Ahmad P., Akram N.A. (2018). Environmental Stresses and Metabolomics—Deciphering the Role of Stress Responsive Metabolites. Plant Metabolites and Regulation Under Environmental Stress.

[47] Thompson J., Stewart C.R., Morris C.J. (1966). Changes in Amino Acid Content of Excised Leaves During Incubation I. The Effect of Water Content of Leaves and Atmospheric Oxygen Level. Plant Physiol..

[48] Zhang H., Murzello C., Sun Y., Kim M.-S., Xie X., Jeter R.M., Zak J.C., Dowd S.E., Pare P.W. (2010). Choline and Osmotic-Stress Tolerance Induced in Arabidopsis by the Soil Microbe Bacillus subtilis (GB03). Mol. Plant Microbe Interact..

[49] Gou W., Tian L., Ruan Z., Zheng P., Chen F., Zhang J., Cui Z., Li Z., Gao M., Shi W. (2015). Accumulation of choline and glycinebetaine and drought stress tolerance induced in maize (Zea mays) by three plant growth promoting rhizobacteria (PGPR) strains. Pak. J. Bot..

[50] Hayat S., Hayat Q., Alyemeni M., Wani A.S., Pichtel J., Ahmad A. (2012). Role of proline under changing environments. Plant Signal. Behav..

[51] Chen T.H.H., Murata N. (2002). Enhancement of tolerance of abiotic stress by metabolic engineering of betaines and other compatible solutes. Curr. Opin. Plant Boil..

[52] Hamilton E.W., Heckathorn S.A. (2001). Mitochondrial Adaptations to NaCl. Complex I Is Protected by Antioxidants and Small Heat Shock Proteins, Whereas Complex II Is Protected by Proline and Betaine1. Plant Physiol..

[53] Perlikowski D., Czyżniejewski M., Marczak L., Augustyniak A., Kosmala A. (2016). Water Deficit Affects Primary Metabolism Differently in Two Lolium multiflorum/Festuca arundinacea Introgression Forms with a Distinct Capacity for Photosynthesis and Membrane Regeneration. Front. Plant Sci..

[54] Michaletti A., Naghavi M.R., Toorchi M., Zolla L., Rinalducci S. (2018). Metabolomics and proteomics reveal drought-stress responses of leaf tissues from spring-wheat. Sci. Rep..

[55] Mekonnen D.W., Flügge U.-I., Ludewig F. (2016). Gamma-aminobutyric acid depletion affects stomata closure and drought tolerance of Arabidopsis thaliana. Plant Sci..

[56] Klem K., Gargallo-Garriga A., Rattanapichai W., Oravec M., Holub P., Veselá B., Sardans J., Peñuelas J., Urban O. (2019). Distinct Morphological, Physiological, and Biochemical Responses to Light Quality in Barley Leaves and Roots. Front. Plant Sci..

[57] Parida A.K., Panda A., Rangani J. (2018). Metabolomics-Guided Elucidation of Abiotic Stress Tolerance Mechanisms in Plants. Plant Metab. Regul. Under Env. Stress.

[58] Hare P., Cress W., van Staden J. (1999). Proline synthesis and degradation: A model system for elucidating stress-related signal transduction. J. Exp. Bot..

[59] Zinta G., AbcElgawad H., Peshev D., Weedon J.T., van den Ende W., Nijs I., Janssens I.A., Beemster G.T.S., Asard H. (2018). Dynamics of metabolic responses to periods of combined heat and drought in Arabidopsis thaliana under ambient and elevated atmospherioc CO2. J. Exp. Bot..

[60] Yang L., Wen K.-S., Ruan X., Zhao Y.-X., Wei F., Wang Q. (2018). Response of Plant Secondary Metabolites to Environmental Factors. Molecules.

[61] Mundim F.M., Pringle E.G. (2018). Whole-Plant Metabolic Allocation Under Water Stress. Front. Plant Sci..

[62] Miura K., Tada Y. (2014). Regulation of water, salinity, and cold stress responses by salicylic acid. Front. Plant Sci..

[63] Shan C., Liang Z. (2010). Jasmonic acid regulates ascorbate and glutathione metabolism in Agropyron cristatum leaves under water stress. Plant Sci..

[64] Mahouachi J., Arbona V., Gómez-Cadenas A. (2007). Hormonal changes in papaya seedlings subjected to prograssive water stress in this halophyte. Plant Growth Regul..

[65] Kumar S., Pandey A.K. (2013). Chemistry and Biological Activities of Flavonoids: An Overview. Sci. World J..

[66] Intergovernmental Panel on Climate Change (IPCC) (2007). Fourth Assessment Report. Climate Change 2007: Synthesis Report.

[67] Nagarajan S., Jagadish S.V.K., Prasad A.H., Thomar A., Anand A., Pal M., Agarwal P. (2010). Local climate affects growth, yield and grain quality of aromatic and non-aromatic rice in northwestern India. Agric. Ecosyst. Environ..

[68] Scafaro A.P., Haynes P.A., Atwell B.J. (2010). Physiological and molecular changes in Oryza meridionalis Ng., a heat-tolerant species of wild rice. J. Exp. Bot..

[69] Xu S., Li J., Zhang X., Wei H., Cui L. (2006). Effects of heat acclimation pretreatment on changes of membrane lipid peroxidation, antioxidant metabolites, and ultrastructure of chloroplasts in two cool-season turfgrass species under heat stress. Environ. Exp. Bot..

[70] Foyer C.H., Noctor G. (2009). Redox Regulation in Photosynthetic Organisms: Signaling, Acclimation, and Practical Implications. Antioxid. Redox Signal..

[71] Suzuki N., Mittler R. (2006). Reactive oxygen species and temperature stresses: A delicate balance between signaling and destruction. Physiol. Plant..

[72] Potters G., Pasternak T.P., Guisez Y., Palme K.J., Jansen M.A.K. (2007). Stress-induced mosphogenic responses: Growing out of trouble?. Trends Plant Sci..

[73] Wedow J.M., Yendrek C.R., Mello T.R., Creste S., Martinez C.A., Ainsworth E.A. (2019). Metabolite, and transcript profiling of Guinea grass (Panicum maximum Jacq) response to elevated [CO2] and temperature. Metabolomics.

[74] Qi X., Xu W., Zhang J., Guo R., Zhao M., Hu L., Wang H., Dong H., Li Y. (2016). Physiological characteristics and metabolomics of transgenic wheat containing the maize C4 phosphoenolpyruvate carboxylase (PEPC) gene under high temperature stress. Protoplasma.

[75] Carrow R.N. (1996). Summer Decline of Bentgrass Greens.

[76] Huang B., Gao H. (2000). Growth and Carbohydrate Metabolism of Creeping Bentgrass Cultivars in Response to Increasing Temperatures. Crop. Sci..

[77] Youngner V.B., Nudge F.J. (1907). Soil Temperature, Air Temperature, and Defoliation Effects on Growth and Nonstructural Carbohydrates of Kentucky Bluegrass1. Agron. J..

[78] Song S.Q., Lei Y.B., Tian X.R. (2005). Proline Metabolism and Cross-Tolerance to Salinity and Heat Stress in Germinating Wheat Seeds. Russ. J. Plant Physiol..

[79] Yue Y., Jiang H., Du J., Shi L., Bin Q., Yang X., Wang L. (2019). Variations in physiological response and expression profiles of proline metabolism-related genes and heat shock transcription factor genes in petunia subjected to heat stress. Sci. Hortic..

[80] Kishor P., Hong Z., Miao G.H., Hu C., Verma D. (1995). Overexpression of [delta]-Pyrroline-5-Carboxylate Synthetase Increases Proline Production and Confers Osmotolerance in Transgenic Plants. Plant Physiol..

[81] Solomon A., Beer S., Waisel Y., Jones G.P., Paleg L.G. (1994). Effects of NaCl on the carboxylating activity of rubisco from Tamarix jordanis in the presence and absence of proline-related compatible solutes. Physiol. Plant..

[82] Prasad K., Saradhi P.P. (1995). Effect of zinc on free radicals and proline in Brassica and Cajanus. Phytochemisty.

[83] Kumar D., Chattopadhyay S. (2018). Glutathione modulates the expression of heat shock proteins via the transcription factors BZIP10 and MYB21 in Arabidopsis. J. Exp. Bot..

[84] Kocsy G., Szalai G., Galiba G. (2002). Induction of Glutathione Synthesis and Glutathione Reductase Activity by Abiotic Stresses in Maize and Wheat. Sci. World J..

[85] Locy R.D., Wu S.-J., Bisnette J., Barger T.W., McNabb D., Zik M., Fromm H., Singh N.K., Cherry J.H. (2000). The Regulation of GABA Accumulation by Heat Stress in Arabidopsis. Plant Tolerance to Abiotic Stresses in Agriculture: Role of Genetic Engineering.

[86] Rao S.R., Ravishankar C. (2008). Enhanced catharanthine and vidoline production in suspension cultures of Catheranthus roseus by ultraviolet-B light. J. Mol. Signal..

[87] Yu K.-W., Murthy H.N., Hahn E.-J., Paek K.Y. (2005). Ginsenoside production by hairy root cultures of Panax ginseng: Influence of temperature and light quality. Biochem. Eng. J..

[88] Chan L.K., Koay S.S., Boey P.L., Bhatt A. (2010). Effects of abiotic stress on biomass and anthocyanin production in cell cultures of Melastoma malabathricum. Boil. Res..

[89] Zobayed S.M.A., Afreen F., Kozai T. (2005). Temperature stress can alter the photosynthetic efficiency and secondary metabolite concentrations in St. John’s wort. Plant Physiol. Biochem..

[90] Singsaas E.L. (2000). Terpenes and thermotolerance of photosynthesis. New Phytol..

[91] Lichtenthaler H.K., Schwender J., Disch A., Rohmer M. (1997). Biosynthesis of isoprenoids in higher plant chloroplasts proceeds via a mevalonate-independent pathway. FEBS Lett..

[92] Henry L.K., Gutensohn M., Thomas S.T., Noel J.P., Duradeva N. (2015). Orthologs of archael isopentenyl phosphate kinase regulate terpenoid production in plants. Proc. Nat. Acad. Sci. USA.

[93] Sairam R., Tyagi A. (2014). Physiology and molecular biology of salinity stress tolerance in plants. Curr. Sci..

[94] Peters G.P., Andrew R.M., Canadell J.G., Friedlingstein P., Jackson R.B., Korsbakken J.I., Le Quéré C., Peregon A. (2019). Carbon dioxide emissions continue to grow amidst slowly emerging climate policies. Nat. Clim. Change.

[95] Duval B.D., Blankinship J.C., Dijkstra P., Hungate B.A. (2011). Retracted Asticle: CO2 effects on plant nutrient concentration depend on plant functional group and available nitrogen: A meta-analysis. Plant Ecol..

[96] Jin J., Tang C., Sale P. (2015). The impact of elevated carbon dioxide on the phosphorus nutrition of plants: A review. Ann. Bot..

[97] Hatfield J.L., Dold C. (2019). Water-Use Efficiency: Advances and Challenges in a Changing Climate. Front. Plant Sci..

[98] Li X., Jal-Ahammed G., Li Z.X., Wei J.P., Shen C., Yan P., Zhang P.P., Han W.Y. (2016). Stimulation in primary and secondary metabolism by elevated carbon dioxide alters green tea quality in Camelia sinensis L. Sci. Rep..

[99] Wenzel S., Cox P., Eyring V., Friedlingstein P. (2016). Projected land photosynthesis constrained by changes in the seasonal cycle of atmospheric CO2. Nature.

[100] Penuelas J., Estiarte M. (1998). Can elevated CO(2) affect secondary metabolism and ecosystem function?. Trends Ecol. Evol..

[101] Penuelas J., Estiarte M., Kimball B. (2000). Flavonoid Responses in Wheat Grown at Elevated CO2: Green Versus Senescent Leaves. Photosynthetica.

[102] Peñuelas J., Fernández-Martínez M., Vallicrosa H., Maspons J., Zuccarini P., Carnicer J., Sanders T.G.M., Krüger I., Obersteiner M., Janssens I.A. (2020). Increasing atmospheric CO2 concentrations correlate with declining nutritional status of European forests. Commun. Boil..

[103] Peñuelas J., Janssens I.A., Ciais P., Obersteiner M., Sardans J. (2020). Anthropogenic global shifts in biospheric N and P concentrations and ratios and their impacts on biodiversity, ecosystem productivity, food security, and human Health. Global Change Biol..

[104] Lindroth R.L., Kinney K.K., Platz C.L. (1993). Responses of deciduous trees to elevated atmospheric CO2, productivity, phytochemistry and insect performance. Ecology.

[105] Penuelas J., Estiarte M., Llusià J. (1997). Carbon-based Secondary Compounds at Elevated CO2. Photosynthetica.

[106] Penuelas J., Llusià J. (1997). Effects of Carbon Dioxide, Water Supply, and Seasonality on Terpene Content and Emission by Rosmarinus officinalis. J. Chem. Ecol..

[107] Penuelas J., Estiarte M. (1996). Trends in plant carbon concentration and plant demand for N throughout this century. Oecologia.

[108] Holopainen J.K., Virjamo V., Ghimire R.P., Blande J.D., Julkunen-Tiitto R., Kivimäenpää M. (2018). Climate Change Effects on Secondary Compounds of Forest Trees in the Northern Hemisphere. Front. Plant Sci..

[109] Koricheva J., Larsson S., Haukioja E., Keinänen M. (1998). Regulation of Woody Plant Secondary Metabolism by Resource Availability: Hypothesis Testing by Means of Meta-Analysis. Oikos.

[110] Sobuj N., Virjamo V., Zhang Y., Nybakken L., Julkunen-Tiitto R. (2018). Impacts of elevated temperature and CO2 concentration on growth and phenolics in the sexually dimorphic Populus tremula (L.). Environ. Exp. Bot..

[111] Vanzo E., Jud W., Li Z., Albert A., Domagalska M.A., Ghirardo A., Niederbacher B., Frenzel J., Beemster G.T., Asard H. (2015). Facing the Future: Effects of Short-Term Climate Extremes on Isoprene-Emitting and Nonemitting Poplar1. Plant Physiol..

[112] Nissinen K., Nybakken L., Virjamo V., Julkunen-Tiitto R. (2016). Slow-growing Salix repens (Salicaceae) benefits from changing climate. Environ. Exp. Bot..

[113] McKiernan A.B., O’Reilly-Wapstra J., Price C., Davies N., Potts B., Hovenden M.J. (2012). Stability of Plant Defensive Traits Among Populations in Two Eucalyptus Species Under Elevated Carbon Dioxide. J. Chem. Ecol..

[114] Randriamanana T.R., Nissinen K., Ovaskainen A., Lavola A., Peltola H., Albrectsen B., Julkunen-Tiitto R. (2018). Does fungal endophyte inoculation affect the responses of aspen seedlings to carbon dioxide enrichment?. Fungal Ecol..

[115] Llusià J., Peñuelas J. (1998). Changes in terpene content and emission in potted Mediterranean woody plants under severe drought. Can. J. Bot..

[116] Peñuelas J., Llusià J. (2003). BVOCs: Plant defense against climate warming?. Trends Plant Sci..

[117] Blanch J.S., Penuelas J., Sardans J., Llusià J. (2008). Drought, warming and soil fertilization effects on leaf volatile terpene concentrations in Pinus halepensis and Quercus ilex. Acta Physiol. Plant..

[118] Bustos-Segura C., Dillon S., Keszei A., Foley W.J., Kulheim C. (2017). Intraspecific diversity of terpenes of Eucalyptus camaldulensis (Myrtaceae) at a continental scale. Aust. J. Bot..

[119] Templer P.H., Pinder R., Goodale C.L. (2012). Effects of nitrogen deposition on greenhouse-gas fluxes for forests and grasslands of North America. Front. Ecol. Environ..

[120] Carter T.S., Clark C.M., Fenn M.E., Jovan S., Perakis S.S., Riddell J., Schaberg P.G., Greaver T.L., Hastings M.G. (2017). Mechanisms of nitrogen deposition effects on temperate forest lichens and trees. Ecosphere.

[121] Schmitz A., Sanders T.G.M., Bolte A., Bussotti F., Dirnböck T., Johnson J., Peñuelas J., Pollastrini M., Prescher A.-K., Sardans J. (2018). Responses of forest ecosystems in Europe to decreasing nitrogen deposition. Environ. Pollut..

[122] Lassaletta L., Billen G., Grizzetti B., Anglade J., Garnier J. (2014). 50-year trends in nitrogen use efficiency of world cropping systems: The relationship between yield and nitrogen input to cropland. Environ. Res. Lett..

[123] Bodirsky B.L., Müller C. (2014). Robust relationship between yields and nitrogen inputs indicates three ways to reduce nitrogen pollution. Environ. Res. Lett..

[124] Larsen S.U., Jorgensen H., Bukh C., Schjoerring J.K. (2019). Green biorefining: Effect of nitrogen fertilization on protein yield, protein extractability and amino acid composition of tall fescue biomass. Ind. Crop. Prod..

[125] Huhn G., Schulz H. (1996). Contents of free amino acids in Scots pine needles from field sites with different levels of nitrogen deposition. New Phytol..

[126] Calanni J., Berg E., Wood M., Mangis D., Boyce R., Weathers W., Sievering H. (1999). Atmospheric nitrogen deposition at a conifer forest: Response of free amino acids in Engelmann spruce needles. Environ. Pollut..

[127] Limpens J., Berendse F. (2003). Growth reduction of Sphagnum magellanicum subjected to high nitrogen deposition: The role of amino acid nitrogen concentration. Oecologia.

[128] Gent M.P.N. (2005). Effect of Genotype, Fertilization, and Season on Free Amino Acids in Leaves of Salad Greens Grown in High Tunnels. J. Plant Nutr..

[129] Ćustić M.H., Horvatić M., Pecina M. (2009). Nitrogen Fertilization Influences Protein Nutritional Quality in Red Head Chicory. J. Plant Nutr..

[130] Kanmegne G., Mbouobda H.D., Fotso-Mbakop C.N., Omokolo D.N. (2017). The influence of stock plant fertilization on tissue concentrations of nitrogen, carbohydrates, and amino acids and on the rooting of leaf stem cuttings of Cola anomala K. Schum (Malvaceae). New For..

[131] Xu Y., Xiao H. (2017). Free amino acid concentrations and nitrogen isotope signatures in Pinus massaniana (Lamb.) needles of different ages for indicating atmospheric nitrogen deposition. Env. Pollut..

[132] Nokerbekova N., Suleimenov Y.T., Zhapayev R. (2018). Influence of Fertilizing with Nitrogen Fertilizer on the Content of Amino Acids in Sweet Sorghum Grain. Agric. Food Sci. Res..

[133] Wen G., Cambouris A.N., Ziadi N., Bertrand A., Khelifi M. (2019). Nitrogen Fertilization Effects on the Composition of Foliar Amino Acids of Russet Burbank Potato. Am. J. Potato Res..

[134] Olsen K.M., Slimestad R., Lea U.S., Brede C., Løvdal T., Ruoff P., Verheul M., Lillo C. (2009). Temperature and nitrogen effects on regulators and products of the flavonoid pathway: Experimental and kinetic model studies. Plant Cell Environ..

[135] Allwood J.W., Chandra S., Xu Y., Dunn W.B., Correa E., Hopkins L., Goodacre R., Tobin A.K., Bowsher C. (2015). Profiling of spatial metabolite distributions in wheat leaves under normal and nitrate limiting conditions. Phytochemisty.

[136] Prinsi B., Espen L. (2015). Mineral nitrogen sources differently affect root glutamine synthetase isoforms and amino acid balance among organs in maize. BMC Plant Boil..

[137] Pietilä M., Lähdesmäki P., Pietiläinen P., Ferm A., Hytönen J., Pätilä A. (1991). High nitrogen deposition causes changes in amino acid concentrations and protein spectra in needles of the scots pine (Pinus sylvestris). Environ. Pollut..

[138] Ann-Brittedfast T.N., Ericsson A., Nordén L.-G. (1994). Accumulation of amino acids in some boreal forest plants in response to increased nitrogen availability. New Phytol..

[139] Cánovas F.M., Ávila C., Cantón F.J.R., Cañas R.A., De La Torre F. (2007). Ammonium assimilation and amino acid metabolism in conifers. J. Exp. Bot..

[140] Nordin A., Näsholm T. (1997). Nitrogen storage forms in nine boreal understory plant species. Oecologia.

[141] Britto D.T., Siddiqi M.Y., Glass A.D.M., Kronzucker H.J. (2001). Futile transmembrane NH4+ cycling: A cellular hypothesis to explain ammonium toxicity in plants. Proc. Natl. Acad. Sci. USA.

