# Supplementary material for: Ecometabolomics for a Better Understanding of Plant Responses and Acclimation to Abiotic Factors Linked to Global Change
Source: Metabolites. 2020 Jun 9;10(6):239. doi: 10.3390/metabo10060239 (PMC7345909; doi:10.3390/metabo10060239)
Supplement: Supplementary file 1 [file metabolites-10-00239-s001.pdf]

Supplementary Material

**Table S1.** Drought impacts on higher plant metabolism.

| Nº | Bibliographic Data | Species (Organs Studied) | Site and Treatment/Gradient Conditions | Metabolic Platform | Statistical Methods | Bioinformatic Tools | Metabolic Pathways and/or Metabolites Up-regulated | Metabolic Pathways and/or Metabolites Down-regulated |
|---|---|---|---|---|---|---|---|---|
| 1 | Foito et al. (2009) Plant Biotechnology Journal | *Lolium perenne* (leaves) | Water manipulation in greenhouse | GC-MS | Multivariate analyses in Genstat version 9.2.0.153 | AMDIS™, XCALIBUR™ | Glucose, raffinose, fructose, trehalose, maltose | Fatty acids |
| 2 | Cramer et al. (2007) Functional Integrative Genomics | *Vitis vinifera* (leaves) | Water manipulation in greenhouse | GC-MS | ANOVAs of all determined metabolites | | Malate, chloride, proline, phosphate, glucose | Succinate, fumarate, aspartate, sucrose |
| 3 | Rivas-Ubach et al. (2012) PNAS | *Erica multiflora* (leaves) | Water supply manipulation in field conditions | 1D and 2D $^1$H NMR spectroscop y | PERMANOVA (PERMANOVA+ for PRIMER v.6). ANOVAs, post hoc tests, PCAs, Kolmogorov–Smirnov tests, and discriminant analyses with Statistica v8.0 (Statsoft) | TopSpin 1.3 (Bruker Biospin), AMIX (Bruker Biospin) | polyphenolic compounds, quinic acid, tartaric acid, and choline | |
| 4 | Rivas-Ubach et al. (2014) New Phytologist | *Quercus ilex* (leaves) | Water supply manipulation in field conditions | LC-MS 1D and 2D $^1$H NMR spectroscop y | PERMANOVA (PERMANOVA+ for PRIMER v.6). PCA and PLS-DA with mixOmics package of R | MZMINE 2.10 | -◎-glucose, sucrose, adenine, polyphenols, phenolic acids, quinic acid, catechin, chlorogenic, epicachetin | -Arginine, alanine, pyridoxine |
| 5 | Gargallo-Garriga et al. (2014) Scientific Reports (2015) | *Holcus lanatus* and *Alopecurus* | Water supply manipulation in semi- | LC-MS | The PCAs were performed with mixOmics package of R. | TopSpin 3.1 software | -Phenilalanine, SA, thymine, adenine, uracil, catequin, galangin (in roots). | -Tartate, pyruvate, Jasmonic acid, indol acatic, |

| | | | | | | | | |
|---|---|---|---|---|---|---|---|---|
| | | *pratensis* (roots and shoots) | natural grassland | | PERMANOVA, PLS, and CIM (clustered image maps) were also conducted with R | | Choline, pyruvate, caffeic acid, quercitin, sabinene, ocimene (shoots) | ocimene, kaemphenol (roots). Valine, tryptophan, threonine, leucine, proline, isoleucine, alanine, glutamic acid, glycine betaine, malic acid, JA, indol acetate, vainillic acid, hexose, manosa, uracil, uridine |
| 6 | Shi et al. (2015) Journal of Experimental Botany | *Cynodon dactylon* (leaves) | Water supply manipulation in greenhouse | GC-TOF-MS | ANOVAs of all determined metabolites comparing control vs treatments | mass spectral libraries (NIST 2005) | -Aspargine, ascorbic acid, galactinol | -Gluconic acid, pantotenic acid |
| 7 | Rivas-Ubach et al. (2016) Perspectives in Plant Ecology, Evolution and Systematics | *Quercus ilex* (leaves) | Water supply manipulation in field conditions | LC-MS | PERMANOVA (PERMANOVA+ for PRIMER v.6). PCA and PLS-DA with mixOmics package of R | MZmine2.12 | -Succinic acid, chlorogenic acid, malic acid, gallic acid, pyruvate, citric acid, hexoses, pentoses, quercitin, tryptophan, a-humulene, luteolin, kaempferol, epigallocatechin, catechin, lactic acid | |
| 8 | Nakabayashi et al. (2014) Plant Signaling Behaviour | *Arabidopsis thaliana* | Water supply manipulation in pot conditions | LC-QTOF-MS | PCA was performed with the SIMCA-P 11.5 software | MassLynx ver. 4.1 (Waters) | -Glycosides of kaempferol, quercetin, and cyanidin, proline, | |

| | | | | | | | | |
|---|---|---|---|---|---|---|---|---|
| | | | | | | | raffinose, galactinol, anthocyanins | |
| 9 | Piasecka et al. 2017 The Plant Journal | *Hordeum vulgare* | Water supply manipulation in pot conditions | LC-MS/MS | ANOVAs of all determined metabolites comparing control vs treatments, PCA with hierarchical bi-clustering of RILs and traits (with the function heatmap2 in R) | Genstat 18 (VSN International, 2015) | -Hydroxycinnamic esters of flavones, 7-O-glycosides and acylated glycosides of flavones, chlorogenic acid | |
| 10 | Ye et al. (2016) Frontieres in Plant Science | *Cynodon dactylon* | Water supply manipulation in pot conditions | GC-TOF-MS | ANOVAs of all determined metabolites comparing control vs treatments (SPSS 13.0 software). Hierarchical cluster analysis was performed using CLUSTER program, and resulting tree | Figures were displayed using the software package and Java Treeview | -Sucrose, soluble sugars, proline | |
| 11 | Gargallo-Garriga et al. (2018) Scientific Reports | *Quercus ilex* (root exudates) | Water supply manipulation in pot conditions | LC-MS | PERMANOVAs and PLSDAs usingthe *mixOmics* package of R | MZMINE 2.10 | -Abcisic acid, leucine, acacetin, malic acid, proline, choline, homoorientin | -Valine, arginine, fructose, ribose, alanine, lysine, asparagine, uracil, glutamine, methionine |
| 12 | Aidoo et al. (2017) Metabolomics | *Triticum aestivum* (different genotypes) (leaves) | Water supply manipulation in pot conditions | GC-MS | Student's *t* test and ANOVAs of all determined metabolites comparing control vs treatments using TMeV statistical software | Xcalibur® | -In the most of the genotypes: Aconitate, ascorbate, galactose, raffinose, maltose, glycine, valine, galactinol, proline, manitol, sorbitol, myo-inositol, glucose, | |

| | | | | | | | | |
|---|---|---|---|---|---|---|---|---|
| | | | | | | | citrate, succinate, asparagine, glutamate, lysine, phenylalanine, ascorbate, proline | |
| 13 | Fischer et al. (2016) Frontieres in Plant Science | *Brachypodium distachyon* (Abovegro und biomass) | Water supply manipulation in pot conditions | FIE-MS | ANOVA, Pearsons correlation analyses, Principal Component Analyses (PCA), and Hierarchical Cluster Analyses (HCA) were performed using R-based MetaboAnalyst 2.0 interface | | -TCA intermediates, alanine and salicylate | |
| 14 | Barchet et al. (2013) Tree Physiology | *Populus* sp. (leaves) | Water supply manipulation in pot conditions | GC-MS | ANOVAs of all determined metabolites comparing control vs treatments | XCMS R-package | -Valine, leucine, proline, isoleucine threonine, phenylalanine, galactinol, catechin, raffinose, tryptophan | -Ketoglutaric acid, succinic acid, fumaric, acid, shikinic acid, malic acid, quinic acid |
| 15 | Alvarez et al (2008) Plant Cell Environment | *Zea mays* (xylem sap) | Water supply manipulation in pot conditions (chamber) | LC-MS/MS | *t*-tests between controls and treatments | | -Glutamine, glycine, serine, threonine, valine, malato, histidine, arginine, leucine, ileucine, asparagine, methionine, phenylalanine, tyrosine, ornithine, proline succinate, tryptophan | -Cytokinin, caffeic acid, ABA, coumarin, cysteine, citrulline |
| 16 | Sanchez et al. (2012) Plant Cell | *Lotus japonica* (leaves) | Water supply manipulation in pot | GC-MS | Student's *t*-test and ANOVA were performe using | NetCDF file format using ChromaTOF software | -Proline, fructose, glucose, maltose, galactiol, succinic | -Aspartic acid, serine, glutamic acid, threonine, |

| | | | | | | | | |
|---|---|---|---|---|---|---|---|---|
| | and Environmental | | conditions (greenhouse) | | multiexperiment viewer software, MeV | | acid, methylmalic acid, saccharic acid, glucuronic acid, gulonic acid, myo-inositol, threonic acid, galactonic acid | glycerophosphoglycerol |
| 17 | Benevenuto et al. (2017) PlosOne | *Zea mays* (leaves) | Water supply manipulation in pot conditions (greenhouse) | LC-MS/MS | ANOVAs of all determined metabolites comparing control vs treatments using | ImageMasterTM 2D Platinum ver. 7.06 | -Silicic acid, methyljasmonate, jasmonic acid, abcisdic acid | |
| 18 | Kang et al (2019) PlosOne | *Triticum aesticum* (leaves) | Water supply manipulation in pot conditions (greenhouse) | GC-MS | PLS-DA using MetaboAnalyst 3.0 server, Analysis of variance with 'lme4' package in R | Automated Mass Spectral Deconvolution and Identification System (AMDIS) (National Institute of Standards and Technology, NIST) | -Proline, tryptophan, valine linoleic acid, , citric acid, fumaric acid, malic acid, fructose, glucose, lysine, isoleucine, leucine, ⊚-tocopherol, galactinol, mannitol, ribitol, tyrosine, gluconic acid, isocitric acid, threonic acid, glycerol sugar | -Aspartic acid, glutamic acid, serine, glycerid acid, sitosterol, putrescine, alanine, glycine, tetradecanol, tetracontanol, benzoacetic acid, hydrocinnamic acid, pyruvic acid |
| 19 | Tschaplinski et al. (2019) Annals of Botany | *Populus deltoids* (leaves) | Water supply manipulation in pot conditions (greenhouse) | GC-MS | PCA using the JMPR, Version 14 (SAS Institute Inc., Cary, NC, USA, *t*-tests between controls and treatments) | | -Coumaroyl glycoside, 5-oxo-proline, ascorbic acid glucoside, salicortin, gallic acid | -Quercitin, galangin, kaempferol, isorhamnetin |
| 20 | Mibei et al. (2017) | *Solanum aethiopicum* (leaves) | Water supply manipulation in pot | GC-MS | ANOVA was conducted using Gen-Stat Discovery (14th edition). PCA was performed | | -Sucrose, fructose, mannose, Xylose, trehalose, isoleucine, | -Maleate, serine, glycerate |

| | | | | | | | | |
|---|---|---|---|---|---|---|---|---|
| | | | conditions (greenhouse) | | using DARwin version 6 software, MetaboAnalyst software, XCMS online (Scripps Research Institute, La Jolla, CA, USA) | | proline, glutamate, fumarate, malate | |
| 21 | Moschen et al. (2017) Plant Molecular Biology | *Helianthus annuus* (leaves) | Water supply manipulation in pot conditions (greenhouse) | GC-MS | ANOVAs of all determined metabolites comparing control vs treatments | | -Glucose, xylose, D-galactinol, raffibose, glucoheptose, fructose, sorbose, glycerate, tyramine, pyruvate, malate, proline, 2-oxoglutarate, myo.inositol | -Tryptophan, homoserine, aspartate, asparagine, ornithine, histidine, lysine |
| 22 | Obata et al. (2015) Plant Physiology | *Zea mays* (leaves) | Water supply manipulation in field | GC-MS | ANOVA with Tukey's Post hoc test, heat map, hierarchical clustering, Bonferroni correction, PCA, box plot, Venn diagram, correlation analysis, and Student's t test) were performed using the R software 3.1.1 | | -Isoleucine, valine, threonine, 4-aminobutanoate, tryptophan, | -Aspartate, maltiol, proline |
| 23 | Moradi et al. (2017) Analytical Biochemistry | *Thymus vulgaris* and *Thymus serpillum* (leaves) | Water supply manipulation in pot conditions (greenhouse) | FI-ICR- MS | PCA was performed using MATLAB software | | -Valine, proline, sorbitol, manitol, xylulose, gluconic acid, choline, ascorbate, sorbitolindole-3-acetaldehyde, coumarin, | -Glutamine, tyrosine, aspartate, alanine |

| | | | | | | | | |
|---|---|---|---|---|---|---|---|---|
| 24 | Pavli et al. (2013) Plant Omics Journal | *Sorghum bicolor* (leaves and roots) | Water supply manipulation in pot conditions (greenhouse) | GC-MS | PCA were performed using SPSS statistical software version 18 (SPSS, 2009). | AMDIS software | -Alanine, aspartic acid, isoleucine, glycine, alanine, asparagine, glutamic acid, homoserine, threonine, tryptophan, valine, tyrosine, caffeic acid, malic acid, fumaric acid, succinic acid, mucic acid, pipecolic acid, saccharic acid, succinic acid, trans-aconic acid, hydroxycinnamic acid, trehalose, fructose, raffinose, sedoheptulose , sucrose | -Talose, D-pantenol, urethane, adenosine, adenine |
| 25 | Xiong et al. (2019) BBA Proteins and Proteomics | *Oryza sativa* (spikes) | Water supply manipulation in common garden conditions | LC-MS | PCA and OPLS-DA using SIMCA software package (version 14.0, Umetrics, Umeå, Sweden) | Metabolomics data were acquired using the software XCMS version 1.50.1 | -Flavonols | |
| 26 | Sanchez-Martin et al. (2015) Plant Cell and Environment | *Avena sativa* (leaves) | Water supply manipulation in pot conditions (greenhouse) | DI-ESI-MS, HPLC-MS/MS | ANOVAs of all determined metabolites comparing control vs treatments by using SPSS software (SPSS Inc., Chicago, IL, USA). PCA and PC-DFA were performed using Pychem 2.0 | | -Glyoxilate, 2-phosphogycolate, ribulose-1,5-bisphosphate | |

| | | | | | | | | |
|---|---|---|---|---|---|---|---|---|
| 27 | Arbona et al. (2010) Journal of Plant Physiology | *Arabidopsis thaliana and Thellungiella halophile* (leaves) | Water supply manipulation in pot conditions (greenhouse) | LC-MS | ANOVAs of all determined metabolites comparing control vs treatments | XCMS with the CAMERA package, Masslynx v4.1 | -ABA, Jasmonate, proline | |
| 28 | Charlton et al. (2008) Metabolomics | *Pisum sativum* L. (leaves) | Water supply manipulation in pot conditions (greenhouse) | $_1$H NMR | Student's t-tests, PCA, PLS-LDA with Matlab | Topspin v 1.3 (Bruker, Germany). | -Proline, valine, threonine, homoserine, myoinositol, ⊚-aminobutyrate, nicotic acid betaine | |
| 29 | Vasquez-Robinet et al. (2008) Journal of Experimental Botany | *Solanum tuberosum* genotypes (leaves) | Water supply manipulation in pot conditions (greenhouse) | GC-MS | PCA using the XLSTAT-Pro (v.7.5.3) program (Addinsoft, NY, USA). | | -Caffeic acid, citric acid, malonic acid, GABA, maltose, trehalose, galactinol, ribose, proline | -Malic acid. Quinic acid, glucose-6-phosphate, trehalose |
| 30 | Evers et al. (2010) Journal of Experimental Botany | *Solanum tuberosum* genotypes (leaves) | Water supply manipulation in field conditions | LC-MS | ANOVAs of all determined metabolites comparing control vs treatments | | -Galactose, inositol, proline, galactinol, ornithine | |
| 31 | Yobi et al. (2013) Molecular Plant | *Saliginella lepidophylla* (whole organism) | Water supply manipulation in growth chamber | GC-MS LC-MS/MS | PCA and PLS-DA were performed using JMP (SAS, www.jmp.com), a commercial software package, and 'R' (http://cran.r-project.org/) | | -Glycolisis/gluconeogenesis and tricarboxylic acid cycle intermediates, nitrogen-rich and ⊚-glutamyl amino acids | -Valine, sugar acids, sugar alcohols |
| 32 | Witt et al. (2012) Molecular Plant | *Zea mays* (leaves and other tissues) | Water supply manipulation in pot conditions (greenhouse) | GC-MS | Student's t-test, ANOVA, Bonferroni-correction, PCA and boxplots were performed using the R- | | -Proline, tryptophan, histidine, phenylalamine, adenine, b-alanine, butanoic acid, | -Ferulic acid |

| | | | | | | | | |
|---|---|---|---|---|---|---|---|---|
| | | | | | software environment 2.13.0 (http://cran.r-project. org/) | | isoleucine, tartronic acid, asparagine, glutamine | |
| 33 | Pinasseau et al. (2017) Frontieres in Plant Science | *Grape vinifera* (fruit) | Water supply manipulation in field | LC-MS | PCA, ANOVA and correlation analyses | | -Resveratrol, hydroxycinnamic acid, benzoic acids, delphinidin, petunidin, malvidin, cyaniding 3,5-diglucoside, petunidin 3,5-diglucoside | -Catechin, flavan-3-ol monomers, catechyl-pyranoanthocyanidins, caffeic acids, total flavan-3-ols, piceatannol |
| 34 | Wedeking et al. (2018) PlosOne | *Beta vulgaris* (leaves and roots) | Water supply manipulation greenhouse | $^{1}$H NMR | ANOVA andPCA wereperformed. A Kaiser-Meyer-Olkin (KMO) value of >0.80 and a significant Bartlett test ($p<0.001$) for sphericity to test the PCA suitability. Statistical, all analyses were performed with SPSS 23.0 (SPSS Inc., New York, USA). | | -Glutamine, tyrosine, tryptophan, leucine, valine, isoleucine, alanine, asparagine, phenylalaninde, pyroglutamate | |
| 35 | Rastogi et al. (2019) PlosOne | *Ocimum tenuiflorum* (leaves) | Water supply manipulation in pot conditions (greenhouse) | GC-MS | ANOVAs of all determined metabolites comparing control vs treatments | | | -Caryophyllene, Methylleugenol |
| 36 | Kang et al. (2019) PlosOne | *Triticum aesticum* (leaves and roots) | Water supply manipulation in pot | GC-MS | The cvs file was uploaded to the MetaboAnalyst 3.0 server | | -Tryptophan, valine, citric acid, fumaric acid, malic acid, Glycerid acid, 3- | -Tryptophan, valine, citric acid, fumaric acid, malic acid (roots) |

| | | | | | | | | |
|---|---|---|---|---|---|---|---|---|
| | | | conditions (greenhouse) | | (http://www.metaboanalyst.ca) for successive analysis, consisting in multifactorial ANOVAs, PLS-DA. Moreover, ANOVA was calculated with assistance of 'lme4' and 'lsmeans' package in R. | | hydroxy propanoic acid, leucine, galactose (leaves) | -Benzenacetic acid (leaves) |
| 37 | Casartelli et al. (2018) Rice | *Oryza sativa* (shoots and roots) | Water supply manipulation in pot conditions (greenhouse) | GC-MS | ANOVAs of all determined metabolites comparing control vs treatments | metabolite networks were constructed using KEGG pathway maps web tool (http://www.genome.jp/kegg/) | -Sucrose, galactinol, rafinosse, myoinositol, tetrahalose, tryptophan, phenylalanine, leucine, valine, isoleucine, threonine, methionine, lysine, asparagine, ornithine, uridine, allantoin, arginene, proline, glutamate, glutamine (shoots and roots),<br>-Glucose, fructose, malonic acid (shoots) | -Malate, arabinose, glucose, fructose, pyridoxamine (roots) |
| 38 | Savoi et al. (2016) BMC Plant Biology | *Vitis vinifera* (fruit) | Water supply manipulation in field | GC-MS<br>LC-MS | ANOVAs of all determined metabolites comparing control vs treatments using JMP 7 (SAS Institute Inc.). PCA was performed using R software. | | -Monoterpenes, benzoic acid, cinnamic acid, zeaxanthin | |

| | | | | | | | | |
|---|---|---|---|---|---|---|---|---|
| 39 | Ullah et al. (2017) BMC Genomics | *Triticum* sp. *Aegilops* sp. (leaves and roots) | Water supply manipulation in pot conditions (greenhouse) | GC-MS | PCA and PLS-DA were performed using Cluster (version 3.0) | The Cytoscape software was used to reveal metabolite-metabolite interaction | -Sucrose, tetrahalose, maltose, proline, glycine, asparagine, methionine, homocysteine, serine, aspartate, alanine, tyrosine, citrate, gluconate, mannitol, valine, gluthatione (leaves and roots), -Glucose, malonic acid, mannose, glyceric acid, galactose, fructose, quinic acid, oxalic acid (leaves) -Oleic acid, ascorbic acid, lysine, leucine, fumaric acid, mandelic acid, cysteine, sikinic acid, malic acid, adipic acid (roots) | Glucose, malonic acid, mannose, glyceric acid, galoactose, fructose, quinic acid, oxalic acid (roots) -Oleic acid, ascorbic acid, lysine, leucine, fumaric acid, mandelic acid, cysteine, sikinic acid, malic acid, adipic acid (leaves) |
| 40 | Mutwaki et al. (2017) BMC Plant Biology | *Calotropis procera* (leaves) | Water supply manipulation in growth chamber | LC-MS | | Clustering was carried using Cluster software package (version 3.0) and analysed using Heatmapper (Heatmapper: web-enabled heat mapping) | -Serine, asparagine, arginine, phenylalanine, choline, tryptophan, glutamine, valine, proline, phenylacetaldehyde, threonine | -Benzoate |

| | | | | | | | | |
|---|---|---|---|---|---|---|---|---|
| 41 | Prinsi et al. (2018) BMC Plant Biology | *Vitis vinifera* (roots) | Water supply manipulation in pot conditions (common garden) | GC-MS | Student's t-test through Statistica software v 8.0 (StatSoft Inc., Tulsa) | Golm Metabolome Database | -Glutamine, tyrosine, isoleucine, alanine, leucine, phenylalanine, GABA, glutamate, valine, threonine, tryptophan, serine, proline, methionine, homoserine, glycine, cysteine, aspartate, arabitol, galactinol, mannitol, inositol, glycerol, erythritol, raffinose, isomaltose, galactose, fructose, sucrose, maltose, glucose, arabinose, tetrahalose, xylose | -Succinic acid, shikinic acid, pyruvic acid, malic acid, fumarate, tartaric acid, threonic acid, lactic acid, 2-oxoglutaric, citric acid, arabinonic acid |
| 42 | You et al. (2019) BMC Plant Biology | *Sesamum indicum* (leaves) | Water supply manipulation in pot conditions (greenhouse) | LC-MS GC-MS | PLS-DA and two paired t test with MetaboAnalyst 4.0 server (http://www.metaboanalyst.ca) | REFINER MS® 10.5 (GeneData, http://www.genedata.com). The pathway analysis was performed using MetaboAnalyst for the identified important metabolites using Arabidopsis thaliana pathway libraries | -GABA, allantoin, proline, arginine, tyrosine, tryptophan, phenylalanine, leucine, valine, isoleucine, asparagine, methionine, threonine, serine | -Malate, shikimate, PEP, pyruvate |
| 43 | Michaletti et al. (2018) Scientific Reports | *Triticum* sp. (leaves) | Water supply manipulation in greenhouse | LC-MS | PCA and PLS-DA | Pathways and networks affected by drought was | -Ribulose-5P, Xyloluse-5P, ribose-5P, lysine, methionine, | -Aconitate, putrescine, spermidine, oxaloacetate, |

| | | | | | | | |
|---|---|---|---|---|---|---|---|
| | | | | | performed by the web-based tool MetPA (Metabolic Pathway Analysis) | homocysteine, malate, tyrosine, phenylalanine, tryptophan, spermine, proline, N-acatyl-ornithine | serine, asparagine, alanine |
| 44 | Sun et al. (2013) POJ | *Zea mays* (leaves and roots) | Water supply manipulation in pot conditions (greenhouse) | GC-MS | PCAs were conducted with SPSS 16.0 software (SPSS Inc, U.S.) | NIST Mass Spectral Library 2002 | -Proline, glycine, serine, tyrosine, threonine, asparagine, valine, maltose (leaves and roots) | |
| 45 | Warren et al. (2012) Metabolomics | *Eucalyptus* sp. (leaves) | Water supply manipulation in pot conditions (greenhouse) | GC-MS | t tests and ANOVA of all determined metabolites comparing control vs treatments | | -Arabitol, arabinose, xylose, galactose, galactiol, galactaric acid, mannose, fructose, quercitol, cyclohexanephenol, malate, mannitol | -Raffinose |
| 46 | Ju et al. (2018) | *Vitis vinifera* (leaves) | Water supply manipulation in pot conditions (greenhouse) | GC-MS | Duncan's multiple range tests with SPSS 19.0 software for Windows (SPSS Inc., Chicago, IL, USA). The heatmap was created by Metabo-Analyst 3.0 | The NIST 2002 mass spectroscopy library (National Institute of Standards and Technology, Gaithersburg, MD, USA) | -2-hexanal, 3-hexanal | |
| 47 | Zhang et al. (2017) | *Caragana korshinskii* (leaves, stem, root) | Water supply manipulation in pot conditions (greenhouse) | GC-MS | PCA and PLS-DA were performed with SIMCA-P 11.5 software package (Umetrics, Umea°, Sweden) | Chroma TOF 4.3X software (www.leco.com) and the LECO/Fiehn Rtx5 database (version Rtx5; LECO) were used for metabolite identification | -Lactobionic acid, D-Talose, raffinose, putrescine, phenylalanine, isoleucine, asparagine | -Oxoproline, aspartic acid, glutamic acid, threonic acid |

| | | | | | | | |
|---|---|---|---|---|---|---|---|
| 48 | Georgii et al. (2017) BMC Plant Biology | *Arabidopsis* sp. (leaves) | Water manipulation in pot conditions (greenhouse) | GC-MS FT-ICR-MS | PCA was done using the prcomp function. For Canonical Correlation Analysis, the rcc function of the mixOmics R package version 5.0-1. Pearson correlation coefficients were computed by cor.test | | -Myo-inositol, glycerol-3-P, flavonoids | |
| 49 | Das et al. (2017) Plants | *Glycine maxima* (leaves) | Water manipulation in pot conditions (greenhouse) | GC-MS LC-MS LC-MS/MS | The statistical significance of the results was evaluated using Welch's two sample t-test | The metabolite clusters were visualized using JAVA TREEVIEW Software. MapMan application software (MapMan Version 3.5.1R2) was used for understanding the metabolic distributions and metabolic regulation in response to the stresses | -Genistin, kaempferol, stigmasterol, daidzein, apigenin, isoliquiritigenin | -Tryptamine, luteolin |
| 50 | Safronov et al. (2017) PlosOne | *Phoenix dactylifera* (leaves and roots) | Water manipulation in growth chamber | GC-MS | ANOVA of all determined metabolites comparing control vs treatments using EdgeR (v 3.14.0) | | -Fucose, glucose, D-Glucopyranose, Galactose, lactose, DL-Glutamine, D-Cellobiose, Arginine, Oxalic acid, Glycine, lyxonic acid, glutamine, sucrose, proline | -Oxalic acid, asparagine, putrescine, lactose |
| 51 | Lenk et al. (2019) International | *Brachypodium* | Water manipulation | LC-MS | heat maps and ANOVA of all determined | | -Lutein, nicotinamide, | -Linolenate, palmitate, |

| | | | | | | | | |
|---|---|---|---|---|---|---|---|---|
| | Journal of Molecular Sciences | *distachyon* (leaf) | in growth chamber | | metabolites comparing control vs treatments | | shikimate-3P, maltose, sphinganine-1P | methylglyoxal, coumaryl, malate, succinate |
| 52 | Correira et al. (2018) Frontieres in Plant Science | *Eucalyptus globulus* (leaves) | Water manipulation in pot conditions (growth chamber) | GC-MS | AOVA, sPLS and PCA using the software R v3.1.2 (R Core Team 2014) core functions plus the package mixOmics v.4.0.2 | | -Malate, citrate, aspartate, glutamate, asparagine, leucine, proline, threonine, lysine, histidine, tryptophan, methionine, GABA, urea, mannose, glucose, maltose, mannitol, sorbitol, inositol | -Succinate, quinate, glycerate, fructose-6P, glucose-6P, dihydroxydrofura none |
| 53 | Li et al. (2018b) Analytical Biochemistry | *Cucumis sativus* (leaves) | Water manipulation in pot conditions (open-top greenhouse) | LC-MS | OPLS-DA and PCA | XCMS online (http://metlin.scripps.e du/çxcms/), for metabolite pathways perform we usedincluding KEGG (http://www.genome.j p/kegg/) and MetaboAnalyst (http://www. metaboanalyst.ca/). | Ileucine, alanine, arginine, sucrose, glucose, trehalose, xylitol, mannose, iditol, raffinose, phenylalanine, proline, tryptophan, abcisic acid, citramalic acid, vanillic acid, phenylpyruvate, hydroxybutiric acid, petrosenilic acid | GABA, threonine, aspartate, Betaine |
| 54 | Tripathi et al. (2016) BMC Genomics | *Glycine max* (leaves and roots) | Water manipulation in a growth chamber | LC-MS/MS GC-MS | t-test was used to identify biochemicals that differed significantly between different time points, treatments and in different tissues | | -Leaves. Glucose, leucine, phenylalanine, tyrosine, tryptophan, valine, threonine, isoleucine, histidine, lysine, homoserine, | |

| | | | | | | | | |
|---|---|---|---|---|---|---|---|---|
| | | | | | | | asparagine, fructose, proline, histidine -Roots. Glycine, serine, glycerate, glucose, sucrose, phenylalanine, tyrosine, tryptophan, proline, histidine, succinate, pipecolate, lysine, aminoadipate, glutarate, 5 aminovalerate, isoleucine, threonine, *b*-alanine, valine, glutarate | |
| 55 | Liu et al. (2019) Planta | *Panicum virgatum* (roots) | Water manipulation in pot conditions (open-top greenhouse) | GC-MS | ANOVA of all determined metabolites comparing control vs treatments | | Arginine, isoleucine, methionine, cysteine, ketose, raffinose, fructose, fucose, sorbose, xylose | |
| 56 | Jia et al. (2019a) Physiologia Plantorum | *Populus deltoids* (leaves) | Water manipulation in pot conditions (greenhouse) | GC-MS | Hierarchical clustering analysis (HCA) of the differentially accumulated metabolites was performed using MeV 4.9 software. non-parametric Mann–Whitney test in SPSS 17.0 program was used to distinguish differentially | | Benzeic acid, ascorbate, a-tocopherol, zeaxanthin, b-carotene, myo-inositol, galactose, lactose, tagatose, fructose, glucose, sucrose, raffinopse, galactinol, tryptophan, salicylic acid, trans-cinnamic acid | Citrate, malate |

| | | | | | | | | |
|---|---|---|---|---|---|---|---|---|
| | | | | | accumulated Metabolites. | | | |
| 57 | Jia et al. (2020) Trees | *Salix sinopurpurea, Salix suchowensis* (leaves) | Water manipulation in a greenhouse | GC-MS | Mann–Whitney test in SPSS 17.0 program was used to distinguish differentially accumulated metabolites. non-parametric Mann–Whitney test in SPSS 17.0 program was used to distinguish differentially accumulated Metabolites. PLS-DA were perfromed using SIMCA-P 12.0 software (Umetrics, Umeå, Sweden) | chromaTOF software (version 4.0) (LECO, St. Joseph, USA) | Saccharic acid, galactose, sedoheptulose, lactose, hydroxycinnamic acid, salicilin, palmitic acid, glycerol, glucose, fructose, tagatose, xylitol, raffinose, mannose, glutamate, lyxose, retinol, threonine, tetrahalose, stearic acid, glucuronic acid, aspartate, xylose, myo-inositol, putrescine, serine, alanine, lactose, mannose, | Malate, succinic acid, citrate, malonic acid, succinate, glycine, pyruvate, threonic acid, shikimate, a-ketoglutaric, glycerate, retinol. |
| 58 | Shahbazy et al. (2020) Plant Science | *Thymus vulgaris* (leaves) | Water manipulation in a growth chamber | FT-ICR-MS | PCA, PLS, HCA, Heatmap, biplot processing, and model validation) were calculated via routines in MATLAB ® R2009a ver. 7.8.0 software (MathWorks© Inc., Natick, MA, USA) | The Metabolite Set Enrichment Analysis (MSEA) software version 3.0 was utilized to identify metabolic pathways (http://www. metaboanalyst.ca/), Xcalibar (Version 2.0.7 Thermo Scientific). data processing in | Glutamate, proline, arginine, glucose, fructose, sucrose, galactose, tagatose, mannose, myo-inositol | Citrate |

| | | | | | | | | |
|---|---|---|---|---|---|---|---|---|
| | | | | | | MATLAB (SIM-stitch algorithm version 2.8), a peak list and peak matrix were generated. | | |
| 59 | Melandri et al. (2020) Journal of Experimental Botany | *Oryza sativa* (leaf) | Water manipulation in a pot experiment (growth chamber) | GC-MS | heat maps, PLSR and PCA and graphical representations were performed using R (version 3.4.3; The R Foundation for Statistical Computing). | ChromaTOF software (LECO) and the library provided by the Golm Metabolome Database (GMD; http://gmd.mpimp-golm.mpg.de/download/) | Alanine, arginine, aspartate, aspartic acid, cysteine, glutamine, glutamic acid, isomaltose, secologanin, glycine, lysine, methionine, serine, threonine, tryptophan, valine, ornithine, tyrosine, raffinose, maltose, myo-inositol, fructose, galactinol, allantoin, valine, phenylalanine, proline, quinic acid, threonic acid, succinic acid, shikimic acid, salicylic acid, | Malic acid, citric acid, isocitric acid, PEP, glyceric acid |
| 60 | Zhang et al. (2014) Plant Cell and Environment | *Medicago trunculata* (roots and shoots) | Water manipulation in a pot experiment (growth chamber) | GC-MS | PCA wasperformed with Spotfire (TIBCO, Somerville, MA, USA) software | | -Roots. Pinitol, proline, malic acid, fructose, myo-inositol<br>-Shoots. Myo-inositol, glucose, fructose, picose, ononotol, ribose, proline. | -Roots. Citric acid, pyroglutamic acid. |

| | | | | | | | | |
|---|---|---|---|---|---|---|---|---|
| 61 | Griesser et al. (2015) Plant Physiology and Biochemistry | *Vitis vinifera* (leaves) | Water manipulation in pot conditions (greenhouse) | GC-MS | Student's t-test by comparing the mean of both groups to detect significantly differing variables betweencontrol and drought stressed plants in R. PCA analyses were performed using the program SPSS for Windows version 16.0 (IBM Corporation, New York, USA). | NIST Chemistry Webbook (http://webbook.nist.gov/ chemistry/). AMDIS software (version 2.65, www.amdis.ne | Succinic acid, tartaric acid, ribose, citric acid, palmitic acid, Coumaric acid, caffeic acid, ferulic acid, resveratrol, epicatchetin, kaemphenol, quercitin, cyanidin, methyl benzene, methyl-butanal, furfural, geranyl acetone | Tetradecanal, pentanal, glycerid acid, malic acid |
| 62 | Silvente et al. (2012) PlosOne | *Glycine max* (leaves) | Water manipulation in pot conditions (greenhouse) | $^1$H NMR | PCAs and ANOVAs were performed using the STATISTICA package for Windows (version 5.1, 1997). | | Succinic acid, aspartate | Glutamine,◎◎-oxoglutarato, GABA, fumaric acid, malic acid, phosphatidycholine, pyruvate |
| 63 | Hochberg et al. (2013) BMC Plant Biology | *Vitis vinifera* (leaves) | Water manipulation in greenhouse | GC-MS | Student's t-test and PCA were performed using R 3.0.1. The Extended Statistics (XS) module of the EZinfo software (Waters LTD) was used to perform OPLS-DA with Pareto scaling. | MassLynxTM software (Waters) version 4.1. The raw data acquired were processed using the MarkerLynx application manager (Waters) | Phenylalanine, leucine, valine, proline, asparagine, threonine, tryptophan | Epicachetin, cachetin |
| 64 | Jia et al. (2016) BMC Genomics | *Astragalus membranaceous mongolicus* (leaves) | Water manipulation in pot conditions | $^1$H NMR | t tests and ANOVA were performed using the SPSS software (version 19, IBM, Armonk, NY, USA). PCA were performed | Chenomx NMR Suite Professional software (version 7.7, Chenomx, Edmonton, Canada). | Sucrose, fructose, glucose, valine, leucine, isoleucine, citrate, proline, fumarate, malate, homoserine | Lactate, Glycerate |

| | | | | | | | | |
|---|---|---|---|---|---|---|---|
| | | | | | using the Chenomx NMR Suite 7.7 software. | | | |
| 65 | Correira et al. (2016) Metabolomics | *Eucalyptus globulus* (leaves) | Water manipulation in pot conditions | GC-MS | ANOVA, sPLS and PCA were performed using the software R v3.1.2 (R Core Team 2014) core functions plus the package mixOmics v.4.0.2 | | Ribulose, fructose, galactose, xylose, myo-inositol, ribose, proline | Glycine, valine, shikimic acid, quercetin, piceatannol, caffeoylquinic acid |
| 66 | Ma et al. (2016) Frontieres in Plant Science | *Oryza sativa* (leaves) | Water supply manipulation in field | GC-MS | PCA) and OPLS-DA in SIMCAP+14.0softwarep ackage (Umetrics, Umeå, Sweden) | ChromaTOF software (v 4.34, LECO, St Joseph, MI). | Alanine, GABA, xylitol, stearic acid, putrescine, malonic acid, saccharic acid, glucose, isoleucine | |
| 67 | Piasecka et al. (2017) The Plant Journal | *Hordeum vulgare* (leaves) | Water manipulation in pot conditions | LC-MS | heatmap2 in R. ANOVA of all determined metabolites comparing control vs treatments. Correlation networks and differential correlation networks were constructed using the WGCNA package in R. | | Ferulic acid, caffeic acid, glucose, hydroxycinnamic acid | |
| 68 | Elber Vital et al. (2017) Plant Molecular Biology | *Saccharum spp* (leaf) | Water manipulation in pot conditions (greenhouse) | GC-MS | ANOVA and Tukey test ($p < 0.05$) was performed using R version 3.1.2 (R Development Core Team 2011). | The metabolite profiling results were visualized in the VANTED software | Glucose, fructose, galactose, xylose, raffinose, phenylalanine, tryptophan, tyrosine, valine, leucine, proline, glutamine, glutamate, methionine, lysine, threonine, isoleucine, | Sucrose, aconitate, isocitrate, pyruvate, alanine |

| | | | | | | | | |
|---|---|---|---|---|---|---|---|---|
| | | | | | | | asparagine, serine, glycine | |
| 69 | Do et al. (2013) PlosOne | *Oryza sativa* (leaves) | Water manipulation in pot conditions | GC-MS | ANOVA of all determined metabolites comparing control vs treatments. | NIST08 software (http://chemdata.nist.gov/) and the mass spectral and retention time index (RI) reference collection of the Golm Metabolome Database | Arginine, GABA, proline, glutamate | Putrescine |
| 70 | Ashrafi et al. (2018) Plant Physiology and Biochemistry | *Thymus vulgaris* (leaves) | Water manipulation in pot conditions (greenhouse) | $^{1}H$ NMR | ANOVA, mean comparison and correlation analysis were performed using R (R Development Core Team, 2008). PCA, hierarchal clustering, and pathway analysis were performed using the Metaboanalyst website | | Sucrose, fructose, glucose, alanine, choline, succinic acid | |
| 71 | Alves Filho et al. (2018) Scientific Reports | *Phyllanthus* sp (leaves) | Water manipulation in greenhouse | $^{1}H$ NMR LC-MS | ANOVA using Origin™ 9.4 software | | Glucose, corilagin, malic acid, succinic acid, citric acid, proline, GABA, alanine, valine | |
| 72 | Acevedo et al. (2019) Planta | *Ilex paraguariensis* (leaf) | Water manipulation in pot conditions | GC-MS | ANOVA and PCA | | Uracil, malate, isomaltose, fucose, asparagine, threonine, methionine, isoleucine, ornithine, | Aspartate, phenolics, citric acid, sucrose, sorbose, raffinose |

| | | | | | | | | |
|---|---|---|---|---|---|---|---|---|
| | | | | | | | glutamine, valine, phenylalanine, serine | |
| 73 | Wang et al. (2019) Acta physiologiae Plantarum | *Glycine soja* (leaves) | Water manipulation in pot conditions | GC-MS | The data were pre-processed using the manufacturer's Chroma TOF software (versions 2.12, 2.22, 3.34; LECO, St. Joseph, MI, USA). ANOVA and PCA were performed in SIMCA-P 13.0 software package (Umetrics, Umea, Sweden). | The metabolic pathways were constructed by KEGG (http://www.genom e.jp/kegg/) and the pathways were analyzed using the MetaboAnalyst website (http://www.metab oanal yst.ca/) | Malic acid, fructose, glucose, gluconic acid, threonine, serine, glycine, valine, proline, isoleucine, phenylalanine, mannose, ribose, xylose, myo-inositol, xylitol, threonic acid | Linoleic acid, glycerol, alanine, asparagine, citric acid, fumaric acid |
| 74 | Pires et al. (2016) Plant Cell and Environment | *Arabidopsis thaliana* (leaves) | Water manipulation in pot conditions | GC-MS | ANOVAs and Student's t-tests performed using the algorithm embedded into Microsoft Excel | | Alanine, aspartate, ◎-alanine, GABA, glutamate, glutamine, guanidine, isoleucine, leucine, lysine, methionine, ornithine, phenylalanine, proline, serine, threonine, tryptophan, valine, 2.-oxoglutarate, fructose, galactose, glucose, maltose, mannose, raffinose, ribose, sucrose, trehalose, cis-aconitate, citrate, dehydroascorbate, | |

| | | | | | | | | |
|---|---|---|---|---|---|---|---|---|
| | | | | | | | fumarate, glycolate, isocitrate, malate, succinate | |
| 75 | Yadav et al. (2019) Journal of Experimental Botany | *Triticum aestivum* (leaves) | Water manipulation in greenhouse | GC-MS | Statistical significances were calculated by two-tailed Welch's *t*-tests in the MetabolomeExpress Comparative Statistics tool. | MetabolomeExpress data processing pipeline (www.metabolome-express.org) | Glycine, threonine, leucine, tryptophan, phenylalanine, glutamine, lysine, methionine, asparagine, GABA | Glycerate, 3-P-glyceraldehide |
| 76 | Rizhsky et al. (2004) Plant Physiology | *Arabidopsis* sp. (leaves) | Water manipulation in growth chamber | GC-MS | ANOVA of all determined metabolites comparing control vs treatments. were done using the SAS system version 8.2 (SAS Institute, Cary, NC) | | Citric acid, proline, fructose, xylitol, galactose, glucose, mannose, sucrose, trehalose | |
| 77 | Cardenas-Manríquez et al. (2016) Environmental and Experimental Botany | *Nicotina tabacum* (leaves) | Water manipulation in pot experiment | GC-MS | ANOVA and Fisher tests. | ChemStation software which was linked to the NIST mass spectral library. To visualize metabolite profile heatmaps were established using the package gplots in R and the web-based pipeline MetaboAnalyst version 2.0 | Anthocyanins, chlorogenic acid, rutin, flavonoids, phenolic acids | |
| 78 | Semel et al. 2007 Metabolomics | *Solanum pennellii* (pericarp) | Water manipulation in field | GC-MS | PCA was performed with the TIGR MultiExperiment Viewer 4.0 and students t-test using the algorithm | | Alanine, GABA, b-alanine, homoserine, isoleucine, proline, serine, valine | Glutamine, glycine, cysteine |

| Nº | Bibliographic data | Species (organs studied) | Site and treatment/gradient conditions | Metabolic platform | Statistical methods | Bioinformatic tools | Metabolic pathways and/or metabolites up-regulated | Metabolic pathways and/or metabolites down-regulated |
|---|---|---|---|---|---|---|---|---|
| | | | | | embedded into Microsoft Excel | | | |
| 79 | Pinheiro et al. (2004) Journal of Plant Physiology | *Lupinus albus* (leaves) | Water manipulation in chamber | $^{1}H$ NMR | ANOVA | | Citrate, malate, sucrose | Proline |

**Table S2.** Heat (warming) impacts on higher plant metabolism.

| Nº | Bibliographic data | Species (organs studied) | Site and treatment/gradient conditions | Metabolic platform | Statistical methods | Bioinformatic tools | Metabolic pathways and/or metabolites up-regulated | Metabolic pathways and/or metabolites down-regulated |
|---|---|---|---|---|---|---|---|---|
| 1 | Du et al. (2011) Physiologia Plantarum | (Kentucky bluegrass) *Poa pratensis*, *Cynodon dactylon* x *Cynodon pratensis* (bermudagrass) | Temperature manipulation in pot conditions (greenhouse) | GC-MS | ANOVA of all determined metabolites comparing control vs treatments using SAS (SAS Institute Inc., Cary, NC) | TURBOMASS 4.1.1 software (PerkinElmer Inc.) coupled with commercially available compound libraries: NIST 2005 (PerkinElmer Inc., Waltham, MS), Wiley 7.0 (John Wiley & Sons Ltd., Hoboken, NJ) | -Malic acid, threonic acid, citric acid, galacturonic acid, gluconic acid, aspartate, proline, ileucine, alanine, serine, glycine, valine, threonine, lysine, phenylalanine, asparagine, methionine, GABA, tyrosine (in bermudagrass)<br>-Threonic acid, citric acid, succinic acid, asparaginelysine-5-hydroxynorvaline (Kentuky bluegrass) | -Methyl malonic acid, glycerid acid (in bermudagrass)<br>-Methyl malonic acid, glycerid acid, succinic acid, ileucine, lysine, glycine, methionine, serine, threonine, valine (Kentuky bluegrass) |
| 2 | Berini et al. (2018) Frontieres in Plant Science | *Abies balsamica*, *Betula* | Temperature manipulation field | LC-MS | PERMANOVA using R 3.5.0 (R Core Team, 2017) | Xcalibur version 2.2 (Thermo Fisher Scientific, Bremen, | -Resin acid (*Abies balsamica*) | -Catechin, terpene acid (*Betula papyrifera*) |

| | | | | | | | | |
|---|---|---|---|---|---|---|---|---|
| | | *papyrifera*, *Corylus avellana*, *Populus tremuloides* (leaves) | | | with the adonis function (vegan package) | Germany) | | |
| 3 | Lei et al. (2018) Journal of Agricultural and Food Chemistry | *Oryza sativa* (fruits) | Temperature manipulation fin phytotron | LC-MS | PCA in the R statistical program (R Core Team, 2013) using the pcaMethods (http://www.bioconductor.org/packages/release/bioc/html/pcaMethods.html) and ggplot2 packages (https://ggplot2.tidyverse.org/) for dimensionality reduction | Progenesis QI (version 2.2) software | -Glutamine, UMP, cytidine, 3,6-Nonadienal, violaxanthin, indole, phenylalanine, tyrosine, p-coumaric acid, p-coumar-dehyde, cellotetraose, 5-oxoproline, leucine, 2-oxolsacaproate, valine, | -Glucose-1-P, linoleate, brassinolide, cycloartenol, undecaprenyl diphosphate |
| 4 | Wedow et al. (2019) Metabolomics | *Panicum maximum* (leaves) | Temperature manipulation field | GC-MS | ANOVA with post-hoc two-tailed Dunnett test using SAS (SAS/STAT v9.4, SAS Institute, Inc.) | AMDIS 2.71 (NIST, Gaithersburg, MD, USA) program | -1,3-diaminopropane, 1-benzylglucopyranoside, g-tocopherol, arabinose, dehydroascorbic acid, fructose, galactose, inositol, isoleucine, leucine, melibiose, O-acetylsalicylic acid, | |

| | | | | | | | | |
|---|---|---|---|---|---|---|---|---|
| | | | | | | | phenylalanine, serine, stigmasterol, threitol, threonine, valine, xylose. | |
| 5 | Wang et al. (2012) Journal of Proteomics | *Glycine maxima* (seeds) | Temperature manipulation in pot conditions (greenhouse) | GC-MS | ANOVA of all determined metabolites comparing control vs treatments | Xcalibur 2.0 software (ThermoFinnigan, Austin, TX, USA) coupled with commercially available mass spectrum libraries: NIST 2005 (Fisons, Manchest, UK) and Wiley 7.0 (Palisade Cooperation, Yonkers, NY). | -Oleic acid, stearic acid | -Linolenic acid |
| 6 | Escandon et al. (2018) Frontieres in Plant Science | *Pinus radiata* (needles) | Pot-chamber experiment | LC-MS | sPLS, PCA and Heatmap clustering with software R v2.15.2 (R Development CoreTeam, 2015) and RStudio (RStudio Team, 2015) | MZmine software version 2.10. Chromatograms were aligned using the RANSAC algorithm. Metabolomics pathways of each metabolite were searched against KEGG pathway maps (KEGG Mapper), and p-values of each metabolomics pathways in MBROLE 2.0 | Phenylalamine, ascorbic acid, glutathione | Phenylpropanoids, flavonols |

| | | | | | | | | |
|---|---|---|---|---|---|---|---|---|
| 7 | Georgii et al. (2017) BMC Plant Biology | *Arabidopsis* sp. (leaves) | Temperature manipulation in pot conditions (greenhouse) | GC-MS FT-ICR-MS | | Mapping of masses to metabolites was performed with MassTRIX and with ChemSpider using the metabolism data sources ChEMBL, BioCyc, AraCyc, MassBank, KEGG and Golm Metabolome Database | -Myo-inositol, glycerol-3-P, flavonoids | -Glycerol, inositol-P |
| 8 | Das et al. (2017) Plants-Basel | *Glycine max* (leaves) | Temperature manipulation in pot conditions (greenhouse) | GC-MS LC-MS LC-MS/MS | MapMan application software (MapMan Version 3.5.1R2) was used for understanding the metabolic distributions and metabolic regulation in response to the stresses | The metabolite clusters were visualized using JAVA TREEVIEW Software. | -Mucic acid, genistin, stignasterol, isorhannetintyranine | -Daidzein, daidzin, formononetin, glycitin, syringic acid, genistein, genistin, adenine, tryptamine, luteolin |
| 9 | Safronov et al. (2017) PlosOne | *Phoenix dactylifera* (leaves and roots) | Temperature manipulation in growth chamber | GC-MS | ANOVA of all determined metabolites comparing control vs treatments using EdgeR (v 3.14.0) | | -Fucose, glucoronic acid, Ornithine-1-5-lactam, Raffinose, Galactinol, malic acid, arginene | -Asparagine, Glucose-1-phosphoric-acid, gliceric acid, Threonic acid-1-4-lactone, fructose, serine, D-Glucopyranose |

| | | | | | | | | |
|---|---|---|---|---|---|---|---|---|
| 10 | Qi et al. (2017) Protoplasma | *Zea mays* (leaves) | Temperature manipulation in pot conditions (phytotron) | GC-MS | ANOVA to analyze treatment effects with Fisher's least significant difference (LSD) test analysis by using SPSS 13.0 software (IBM, Chicago, IL, USA) | Gaphs were plotted using Excel 2003 (Microsoft, USA). | -Citric acid, fumaric acid, hexadecanoic acid, nicotinic acid, octadecanoic acid, succinic acid, threonic acid, asparagine, aspartic acid, glutamic acid, isoleucine, lysine, phenylalanine, proline, serine, valine, fructose, gentiobiose, glucose, xylitol | -Glyceric acid, malic acid, glycine, galactose, melibiose, inositol, mannitol. |
| 11 | Michailidis et al. (2019) Planta | *Prunus mahaleb* (fruit) | Temperature manipulation in common garden | LC-MS/MS | Anova with Duncan's Multiple Range Test. (MANOVA and Spearman correlation analysis were done with SPSS (SPSS v21.0., Chicago, USA). | | -Fructose, glyceril glycoside, leucine, serine, tyrosine, tryptophan, esculetin, phlorizin, kaempferol-3-rutinoside, naringenin-7-glucoside, petunidin, peonidin, cyanidin | -Fructose, , mannitol, xylitol, arabitol, asparagine, glutamate, threonine, tartarate, valinin, cryptochlorogenic acid, quercitin, arbutin |
| 12 | Shen et al. (2019) Tree Physiology | *Camelia sinensis* (leaves) | Temperature manipulation in pot conditions (growth chamber) | LC-MS/MS | PLS-DA and OPLS-DA were conducted using the package ropls (version 1.4.2) under R package (version 3.0.3, www.r-project.org) | Online MWDB (metware database from Metware Biotechnology Co., Ltd, Wuhan) (Chen et al. 2013), MassBank (http://www.massb | -Kuromanin, cyaniding O-hexosyl-O-hexosyl-O-hexoside, cyaniding O-syringic acid, kuromanin chloride, cyaniding 3-galactoside, N-acatylmannosamine | -Dihydroquercitin, pentamethoxuchalcone, O-acetyhexoxide, quercitin |

| | | | | | | | | |
|---|---|---|---|---|---|---|---|---|
| | | | | | | ank.jp/), KNAPSAcK (http://kanaya.naist .jp/KNApSAcK/), HMDB (http://www.hmdb. ca/) (Wishart et al. 2013), MoTo Database (http://www.ab.wu r. nl/moto/) and METLIN (http://metlin.scrip ps.edu/index.php) (Schultz et al. 2013) databases | | |
| 13 | Ren et al. (2019) Forests | *Populus tomentosa* (leaves) | Temperature manipulation in pot conditions (growth chamber) | GC-MS | Student's t-test and PCA with SPSS, (Chicago, IL, USA | Chroma TOF 4.3X software and the LECO-Fiehn Rtx5 database (LECO Corporation, St. Joseph, MI, USA) | Galactinol, raffinose, melibiose, serine, leucine, proline | Myo-inositol, pyruvate, succinate, fumarate, citruline |
| 14 | Serrano et al. (2019) Scientific Reports | *Arabidopsis thaliana* (abovegroun d) | Temperature manipulation in pot conditions (growth chamber) | LC-MS/MS | Matched pairs *t*-test (one sample *t*-test), one-way ANOVA and two-way ANOVA with R (http://cran.r-project.org/) and JMP (SAS, http://www.jmp.c om) | METABOLON, Inc. (North Carolina, USA) | -N-acetylmethionine, 2-isopropylmalate, putrescine, a-ketoglutarate, fumarate, galactinol, raffinose, staschyose, 2-hydroxylaurate, oleylcholine, glycerol-3-P, glycerolphosphoetha nolamine, delta- | -N-acatylserine, tyrosine, N-acetylphenylanine, N-formylphenylalanine, N-acetylthreonine, N-acetylglutamine, N-acetylleucine, sucrose, glunonate, 3-hydroxybutyrate, undercanadioate, 1-linolenoyll-GPC, |

| | | | | | | | | |
|---|---|---|---|---|---|---|---|---|
| | | | | | | | tocopherol, N-carbomoylaspartate, narigenin, sinapate, lariciresinol, sucrose, glycerol-3-p, adenitylsuccinate | methyladenosine, O-methyluridine, guanylyluridine, uridyylguanisine |
| 15 | Wang et al. (2018) Journal of Experimental Botany | *Triticum aestivum* (grain) | Temperature manipulation in pot conditions (greenhouse) | LC-MS/MS | | Chroma TOF 4.3X software (Leco Corp.) and the Leco-Fiehn Rtx5 Database. Enrichment analysis was performed using the software Metaboanalyst 2.0 | -Histidine, glycine, tryptophan, ileucine, cysteine, aspartate, alanine, valine, tyrosine, lysine | -Asparagine, serine |
| 16 | Correira et al. (2018) Frontieres in Plant Science | *Eucalyptus globulus* (leaves) | Temperature manipulation in pot conditions (growth chamber) | GC-MS | AOVA, sPLS and PCA using the software R v3.1.2 (R Core Team 2014) core functions plus the package mixOmics v.4.0.2 | | -Citrate, aspartate, glutamate, isoleucine, proline, phenylalanine, tryptophan, urea, mannitol, sorbitol, inositol | -Malate, glycerate, fructose-6P, dihydroxydihydrofuran one |
| 17 | Koscielny et al. (2019) Crop Pasture Science | *Brasica napus* (flowers) | Temperature manipulation in growth chamber | GC-MS | Mixed lineal model with treatment as fixed effects, and season, genotype, as random effects along with replicate, which was nested within treatmentusing | ChromaTof software version 4.50.8.0 (LECO) and Refiner MS software version 8.1 (Genedata, Basel, Switzerland) | -Pyruvate, haxanoic acid, 2-hydroxyglutaric acid, fructose, raffinose, sucrose, adenosine | -◎-sitosterol, ethanolamine, serine, galactinol, pyroglutamic acid, myo-inositol, aspartic acid, p-coumaric acid, glutamine, digalactosyl glycerol, valine |

| | | | | | | | | |
|---|---|---|---|---|---|---|---|---|
| | | | | | Excel 2010 and R (R Foundation for Statistical computing, Vienna). | | | |
| 18 | Rizhsky et al. (2004) Plant Physiology | *Arabidopsis* sp. (leaves) | Temperature manipulation in growth chamber | GC-MS | ANOVA of all determined metabolites comparing control vs treatments. were done using the SAS system version 8.2 (SAS Institute, Cary, NC) | | -Hydroxysuccinic acid, lactiol, fucose, melibiose | |
| 19 | Kaplan et al. (2004) Plant Physiology | *Arabidopsis* sp. (leaves) | Temperature manipulation in growth chamber | GC-MS | ANOVA was done using the Kruskal-Wallis post-hoc test and PCA were performed with the S-Plus 2000 software package standard edition release 3 (Insightful, Berlin) | | -Uracil, quninic acid, mannose, myo-inositol, shikimic acid, aminovalonic acid, bibonic acid, xylitol, x-ketoglutaric acid, alanine, citric acid, erythirol, erythronic acid, fructose, fumaric acid, GABA, galactinol, galactonic acid, glycerol, glycine, alanine, asparagine, glycerol, homoserine, ileucine, leucine, lysine, methionine, | -Citramalic acid, glycine, glycerid acid, aspartate |

| | | | | | | | | |
|---|---|---|---|---|---|---|---|---|
| | | | | | | | threonine, tyrosine, valine, malic acid, maltose, melibiose, myoinositol, ornithine, putrescine, raffinose, ribose, succinic acid, sucrose, succinic acid, threonic acid, trehalose, tyramine, xylulose | |
| 20 | Austen et al. (2019) Frontieres in Plant Science | *Salix* sp (leaves) | Temperature manipulation in growth chamber | GC-FID GC-MS | A two-way ANOVA with *Post Hoc* Tukey Using the Minitab® Version 17 (Minitab Inc, 2010) Software Package was used to determine whether there was a difference in total isoprene in each sample in relation to the treatments. OPLS-DA and PCA using the SIMCA (Umetrics) statistical package | Metlin (https://metlin.scripps.edu) and Kegg (http://www.genome.jp/kegg/pathway.html) databases | -P-coumaryol-CoA, Cinnamoyl-CoA, pyruvate, glyceraldehyde-P, isopenthenyl-PP, dimethylallyl-PP, isoprene, geranyl-PP | -Quercitin, apigenin, cyaniding, luteolin, kaempferol, naringenin |
| 21 | Raval et al. (2018) Plant Growth Regulation | *Arachis hypogaea* (leaves) | Temperature manipulation in pot conditions (greenhouse) | LC-MS/MS GC-MS | Data processing and statistical analysis of untargeted | Metabolites were putatively identified by matching their | -Putrescine, galactose, threonine, hexopyranose, stearic acid, | |

| | | | | | | | | |
|---|---|---|---|---|---|---|---|---|
| | | | | | metabolites was carried out using MetaboAnalyst 3.0 | mass spectra to spectra in NIST 14 library (National Institute of Standards and Technology, Gaithersburg, MD, USA). Baseline correction, alignment, peak picking, and integration were performed using the ACD/Spec Manager v.12.00 (Advanced Chemistry Development, Inc., ACD/Labs, Toronto, Canada) | Ketoglucose, gulose, serine, catechin, epicatechin | |
| 22 | Glaubitz et al. (2016) Plant Cell Environment | *Oryza sativa (leaves)* | Temperature manipulatuion in chamber experiment | GC-MS | PCA was performed using the R (www.r-project.org) pcaMethods package version 1.64.0. Correlation analysis of metabolite pools with the HNT sensitivity rank was performed with SigmaPlot | Visualization and data-mining software MultiExperiment Viewer (www.tm4.org/mev; version 4.5.1) | Arabinose, fructose, glucose, rhamnose, asparagine, leucine, methionine, phenylalamine, threonine, aspartic acid, salicylic acid, putrescine, saccharic acid, galactiol, sorbitol, pantothenic acid | Sucrose, ascorbic acid, citric acid, isocitric acid, pyridine, glyceric acid |

| | | | | | | | | |
|---|---|---|---|---|---|---|---|---|
| | | | | | 12.3 using Spearman’s rank correlation with Benjamini–Hochberg correction. The cluster analysis and visualization of multivariate intensity profiles were carried out in MarVis-Cluster | | | |

**Table S3.** CO2.

| Nº | Bibliographic data | Species (organs studied) | Site and treatment/gradient conditions | Metabolic platform | Statistical methods | Bioinformatic tools | Metabolic pathways and/or metabolites up-regulated | Metabolic pathways and/or metabolites down-regulated |
|---|---|---|---|---|---|---|---|---|
| 1 | Noguchi et al. (2018) Plant Cell and Environment | *Oryza sativa* (leaves) | FACE experiment | LC-MS | Generalized linear mixed model in lme4 packagewith the statistical software R (R Core Team 2016). | | Alanine, asparagine, histidine, tryptophan, glucose | 2-oxoglutarate |
| 2 | Geng et al. (2016) The Plant Journal | *Brassica napus* (leaves) | Open chamber experiment | LC-MS | Linear models (ANOVA) using R version 3.3.2 (R Development Core Team, 2015). | mzMINE software version 2.1. Plant-Cyc, KEGG, LipidMaps, ChemSpider, METLIN, and PubChem databases | Fructose, deoxyguanosine, guanosine, guanidine, xanthine, hypoxanthine, riboflavin, cysteine, histidine, lysine, asparagine, masaconic acid, | Mannitol, adenosine, histamine, naringenin, isoliquiritigenin, salicin, orotate, inosine |

| | | | | | | | | |
|---|---|---|---|---|---|---|---|---|
| | | | | | | | oxaloacetate, malate, fumarate, succinate, cis-aconitate, shikimate, tryptophan, IAA, lactose, galactose, betaine, sarcosine, dimethylglicine, glutamine, citramalate, pyroglutamate, cyanidin, apigenin, luteolin, quercitin, esculetin, malonate, thymine, uridine, glutamate, pyroglutamate, cytidine, vanillite, alanine, threonine, leucine, isoleucine, galactinol, lactose, JA | |
| 3 | Fernández de Simon et al. (2018) Plant Physiology and Biochemistry | *Pinus pinaster* (leaves) | Pot experiment in grown chamber | GC-MS LC-MS | ANOVAwith Student Newman–Keuls post-hoc testsby using SAS program (version 9.3; SAS Institute, Cary, NC). OPLS-DA (Software SIMCA-13.0, Umetrics AB, Sweden) | MS libraries (Agilent Fiehn GC-MS Metabolomics RTL Library, Wiley7/Nist05 GC/MS Libraries. Metabolite mapping was performed into general biochemical pathways according to Kyoto Encyclopaedia of | Malic acid, glycolic acid, oleic acid, linoleic acid, linolenic acid, sinapoyl desoxyhexose, cumaroyl quinic acid, quercitin, astragalain, levopimaral, kaemphenol-O-hexoside | Oxalic acid, myo-inositol, pinitol,glycolic acid, oleic acid, linolenic acid, shikinic acid, ◎-humulene |

| | | | | | | | |
|---|---|---|---|---|---|---|---|
| | | | | | | Genes the and Genomes (KEGG, http://www.genome.jp/kegg/), the Human Metabolome Database (HMDB, http://www.hmdb.ca/), PubChem project (PubChem, http://pubchem.ncbi.nlm.nih.gov/), and MetaCyc Metabolic Pathway Database (MetaCyc, http://metacyc.org/) | |
| 4 | De Souza et al. (2015)Plant Physiology | *Shorgum bicolor* (leaves, roots and grain) | Pot experiment in grown chamber | LC-MS/MS | Student's t test (n = 3) using JMP Statistical Discovery Software, version 5.0.1. PCA using the softwareMinitab version 14.1. Heat maps were generated usingMetaboAnalyst version 3.0 | | -Grain. Cysteine, pyruvate, phosphoenolpyruvate, fructose, glucose, sucrose, glycine, mevalonate, tryptophan, leucine, phenylalalanine, proline, ADP, histidine, threonine, methionine, sorbitol, alanine, inositol, shikimate, UMP -Leaves. Homoserine, arginine, asparagine, histidine |

| | | | | | | | | |
|---|---|---|---|---|---|---|---|---|
| | | | | | | | -Roots. GTP | |
| 5 | Creydt et al. (2019) Journal of Plant Physiology | *Sambucus nigra* (fruits) | Greenhouse | LC-MS/MS | PCA and PLS-DA | DataAnalysis software (ver. 4.1, Bruker Daltonik GmbH), ProfileAnalysis software (ver. 2.1, Bruker Daltonik GmbH) | Procyanidin | Kaemphenol derivate, petunidin-aldopentose-dihexoside, quercetin-aldopentose-hexoside, cyaniding-desoxyhexose-hexoside |
| 6 | Austen et al. (2019) Frontieres in Plant Science | *Salix* spp. (leaf) | Chamber experiment | GC-MS/MS | A two-way ANOVA with *Post Hoc* Tukey Using the Minitab® Version 17 (Minitab Inc, 2010) Software Package was used to determine whether there was a difference in total isoprene in each sample in relation to the treatments. OPLS-DA and PCA using the SIMCA (Umetrics) statistical package | Metlin (https://metlin.scripps.edu) and Kegg (http://www.genome.jp/kegg/pathway.html) databases | Cinnamoyl-CoA, p-Coumaroyl-CoA, pyruvate, dimethylallyl-PP, Isopentenyl-PP, isoprene | Luteolin, naringenin, dihydrokaemphenol, kaemphenol, quercitin, apigenin, cyanidin |
| 7 | Aranjuelo et al. (2015) Plant Cell and Environment | *Triticum durum* (leaf) | Greenhouse | GC-MS | Pair-wise crossed Student–Welsh, ANOVA, and OPLS-DA carried out with Simca® (MKS Umetrics, Malmö, Sweden). | LECO Pegasus software | Caffeic acid, quinate, aconitate, malate, citrate, succinate, citrate, threonic acid, glucose, fructose, oxoglutarate | Lysina, tryptophan, glycine, serine, GABA, proline |

| | | | | | | | | |
|---|---|---|---|---|---|---|---|---|
| 8 | Aranjuelo et al. (2015) J Experimental Botany | *Nicotiana tabacum* L. (leaves) | Pot-Phytotron | GC-MS | two-factor ANOVA with post-hoc test (LSD) in SPSS v.12.0 ( SPSS Inc., Chicago, IL, USA). | | Maleate, proline, quinate, glucose, glycerol, myo-inositol, galctonate, sucrose | GABA, glutamine, isoleucine, phenylalamine, putrescine, ribose, fructose, mannose, talose, serine, threonine, glycine, methylmaleate, glycerate, sorbitol, xylose |
| 9 | Högy et al. (2010) Journal of Cereal Science | *Triticum aesticum* (grain) | FACE | GC-MS | ANOVA using SPSS PCþ (version 15.0 for windows) | library NIST 2005 (NIST, Gaithersburg, USA) and the Golm metabolome database | Spermidine, ribose, pyruvate, glucuronic acid | Norleucine, aspartate, proline, homoserine, arginine, ornithine, cysteine, tyrosine, Ketoaminobutyrate, gluconate, myo-inositol, glycerol, pyrate, glucuronic acid |
| 10 | Li et al. (2008) Plant Cell and Environmental | *Arabidopsis thaliana* (leaves) | FACE | GC-MS | ANOVA using Microsoft Excel 2002 (Microsoft Corporation, Seattle, WA, USA). PCA implemented in XLSTAT-2007 (Addinsoft, New York, NY, USA) | HP Chemstation (Agilent, Palo Alto, CA, USA) and AMDIS (NIST, Gaithersburg, MD, USA) programs | *Citrate, isocitrate, malate, succinate, tryptophan, phenylalanine, fructose, glucose, maltose, galactose, raffinose, histidine, lysine, methionine* | *Glycine, leucine, valine, asparagine, aspartate, isoleucine, threonine, proline* |
| 11 | Wedow et al. (2019) Metabolites | *Panicum maximum* (leaves) | FACE | GC-MS | ANOVA with post-hoc tests using a two-tailed Dunnett, comparing each treatment to the | AMDIS 2.71 (NIST, Gaithersburg, MD, USA) program | 1-Benzylglucopyranoside, isoleucine, quinic acid, glycerid acid | 2-Methylmalic acid, ◎-cetoglutaric acid, |

| | | | | | | | |
|---|---|---|---|---|---|---|---|
| | | | | control all therm using SAS (SAS/STAT v9.4, SAS Institute, Inc.) | | | |
| 12 | Zhuang et al. (2019) | *Poa pratensis* (leave and roots) | Pot experiment in a growth chamber | GC-MS | ANAOVA with least square differences (LSD) using SPSS 13.0 (SPSS Inc., Chicago, IL, USA). | NIST MS spectral search libraries (National Institute of Standards and Technology, NIST MS search 2.2). | -Leaves: Glucose, sucrose, tagalose, -Roots: Sucrose | -Leaves: Celobiose, galactinol, mucic acid, linoleic acid, -Roots: b-sitosterol, campesterol, stigmasterol, galacturonic acid, palmitic acid, linolenic acid, gluconic acid, succinic acid, turanose, sucrose |
| 13 | Prins et al. (2011) Plant, Cell and Environment | *Zea mays* (leaves) | Chamber experiment | GC-MS | Student's *t*-test or least significant difference (LSD) test | | Galactose, glucose, linolenic acid, linoleic acid, mannose | 2-hydroxyglutaric acid, alanine, 2-ketoglutarate, glutaric acid, m-hydroxybenzoic acid, myo-inositol, p-hydroxybenzoic, proline |
| 14 | Li et al. (2018) Analytical Biochemistry | *Cucumis sativus* (leaves) | Pot-chamber experiment | LC-MS | OPLS-DA and PCA withXCMS online (http://metlin.scripps.edu/xcms/) | XCMS online (http://metlin.scripps.edu/xcms/) | Ribose, monomethyl glutaric acid, Quinovose, Citramalic acid, hydroxyl phennethylalamine, tryptophan, propylene glycol, petroselinic acid, glutathione, | Oxoretinol, ethanolamine, altretamine, hydroxyisovaleric acid, tryptophan, linolenic acid, guanosine, methylophenylacetic acid, GABA, butyrolactone, anisic |

| | | | | | | | | |
|---|---|---|---|---|---|---|---|---|
| | | | | | | | pipecolic acid, thioadenosine, raffinose, pyridoxiamine-5P, iditol, fucose, trehalose, phenylalanine, sucrose, cytosine, mannose, isoleucine, alanine, arginine, vanillic acid, citraconic acid, hydroxybutyric acid, abcisic acid, aminopterin, phenylpyruvate, stachytose, hydroxycinnamic acid, glucose, tagatose, methylmalonic acid, stachytose, proline, xylitol | acid, betaine aldehyde |
| 15 | Levine et al. (2008) Advances in Space Research | *Triticum aesticum* (leaves) | Pot-chamber experiment | GC-MS | ANOVA based on the SAS. PCA was performed with the S-Plus 2000 software package (Insightful, Berlin Germany) | MassLab version 1.4 software (ThermoQuest). AMDIS and NIST02 | Sucrose, fructose, glucose, flavonoids | |
| 16 | Miyagi et al. (2011) Metabolomics | *Rumex obtusifolius* (leaves) | Pot-chamber | CE-MS | Using Microsoft Excel 2003 and Visual Basic 6.0, the heatmap | For visualization analyses employed the Statistical | Citrate, succinate, phenylalanine, isocitrate, shikimate, malate, aconitate, | Ornithine |

| | | | | | | | | |
|---|---|---|---|---|---|---|---|---|
| | | | | | were developed. Correlation and the Student t test analysis were obtained by SPSS. . | Package for the Social Sciences (SPSS v10.0). | fumarate, coumarate, oxaloacetate, pyruvate, ascorbate, PEP, glyceraldehyde, glicyne, citruline, alanine, valine, cinnamate, methionine, GABA | |

**Table S4.** N-deposition (loads).

| Nº | Bibliographic data | Species (organs studied) | Site and treatment/gradient conditions | Metabolic platform | Statistical methods | Bioinformatic tools | Metabolic pathways and/or metabolites up-regulated | Metabolic pathways and/or metabolites down-regulated |
|---|---|---|---|---|---|---|---|---|
| 1 | Freitag et al. (2012) Physiologia Plantarum | Cladonia portentosa (aboveground) | N-deposition gradient | LC-MS | Simple regression analysis (trend) was performed on normalized data in Excel 2003. Spearman's rank order correlation test and ANOVA were perfromed using SPSS | DATAANALYSIS 3.4 Windows 2000/Windows XP application (Bruker Daltonics, Billerica, MA), PROFILEANALYSIS 1.0 (Bruker | -Several fatty acids | |

| | | | | | | | | |
|---|---|---|---|---|---|---|---|---|
| | | | | | 14.0 (SPSS Inc., Chicago, IL). PCA and PLSR | Daltonics). 'SmartFormula Manually' option in DATAANALYSIS 3.4. | | |
| 2 | Gargallo-Garriga et al. (2017) PlosOne | *Heisteria concinna*, *Tetragostris panamensis*, *Alseis blackiana* (leaves) | Field N-fertilization experiment | LC-MS | PERMANOVA, PCA, ANOVA with Bonferroni post hoc test with the *mixOmics*package of R. | Hellinger transformation of data | - *Heisteria concinna*.<br><br>- *Tetragostris panamensis*. Serine<br><br>- *Alseis blackiana*. Pyruvate, uracil, ribose, dissacarid, hexose, manitol, sorbitol, ribose, glutamine, pyruvate, leucine | - *Heisteria concinna*. Threonine, caffeic acid<br><br>- *Tetragostris panamensis*. Adenosine, choline<br><br>- *Alseis blackiana*. Aspartate |
| 3 | Feild and Lake (2011) Physiologia Plantarum | *Carex caryophyllea* genotypes 06, 07, 12 | Pot-growth chamber experiment | TOF-MS | PCA using SIMCA-P (Umetrics, Umea, Sweden) | MASSLYNX (Waters, Milford, MA,) software | -06. Glutamate, Cystenil-glycine<br><br>-07. Glutamate, Cystenil-glycine | |

| | | | | | | | | |
|---|---|---|---|---|---|---|---|---|
| | | | | | | | -12. Glutamate, Cystenil-glycine | |
| 4 | Paudel et al. (2016) BMC Plant Biology | *Arabidopsis thaliana* (leaves) | Pot-growth chamber experiment | LC-MS | PCA and Heatmaps with MetaboAnalyst 3.0 (www.metaboanalyst.ca) | MzMine program (version 2.10). | Glucoerucin | Jasmonic acid, 7-jasmonyl-isoleucine, glucoiberin |
| 5 | Huhn and Schulz (1996) New Phytologist | *Pinus sylvestris* in different stands (needles) Four studies | Field gradient study | LC-FD | U-test of Mann & Whitney was calculated by using the statistical package SPSSPC + 4-0 (SPSS Inc., Chicago, USA). | | -Study 1. Glutamate, aspartate, glutamine, arginine, proline, serine, asparagine, ornithine. -Study 2. Glutamate, glutamine, arginine, proline, serine, asparagine, ornithine. -Study 3. Glutamate, aspartate, glutamine, arginine, proline, | |

| | | | | | | | | |
|---|---|---|---|---|---|---|---|---|
| | | | | | | | serine, asparagine, ornithine, glycine.<br><br>-Study 4. Glutamate, aspartate, glutamine, arginine, proline, serine, asparagine, glycine. | |
| 6 | Lang et al. (2019) Plant Physiology and Biochemistry | *Vitis vinifera* (leaves) | Different field experiments | LC-MS | A MIXED MODEL with a Kenward-Roger test, heatmaps and ANOVA with Tukey HSD test (posthoc test) by using Compound Discoverer 3.0 Software (Thermo Fischer Scientific, Waltham, MA, USA. | | -Study 1. Phenolic compound<br><br>-Study 2. Phenolic compound<br><br>-Study 3. Phenolic compound | -Study 1. Phenolic compound, Kaemphenol -di-hexoside, Kaemphenol -diglucoside, quercitin-di-galactoside, quercitin-diglucoside-galactoside, quercitin-hexoside-glucuronide, kaemphenol-tri-glucoside<br><br>-Study 2. Phenolic |

| | | | | | | | | |
|---|---|---|---|---|---|---|---|---|
| | | | | | | | | compound, Kaemphenol -di-hexoside, Kaemphenol - diglucoside, quercitin-di-galactoside, quercitin-diglucoside-galactoside, kaemphenol-tri-glucoside<br><br>-Study 3. Kaemphenol -di-hexoside, Kaemphenol - diglucoside, kaemphenol-tri-glucoside |
| 7 | Heyneke *et al*. (2017) Frontieres in Plant Science | *Triticum aesticum* (leaves) | Field experiment | GC-MS | Pearson correlation analysis, PCA and ANOVA were performed using R statistical language (http://www.r-project.org/) | TagFinder software | Choline, aspartate, glutamate, serine, glycine, glutamine, valine, isoleucine, tyrosine, leucine, phenylalanine , lysine, | 1-Ketose, shikimate, quinic acid, galactiol, xylose, sorbitol, ribulose, fucose, lyxose, erythriol, isocitrate |

| | | | | | | |
|---|---|---|---|---|---|---|
| | | | | | | histidine, arginine, tryptophan, asparagine, Cis-aconitate, erythromate, alanine |
| 8 | Hu et al. (2019) Plant Physiology and Biochemistry | *Populus* sp (leaves) | Pot-chamber experiment | LC-MS/MS | ANOVA, followed by Tukey's HSD test performed using Excel software (Microsoft, Redmond, WA, USA), and IBM SPSS Statistics 20.0 (StataCorp, College Station, TX, USA). | Glutamine, glutamate, asparagine, oxalacetate, citric acid |

## References


1. Acevedo, R. M.; Avico, E. H.; Gonzalez, S.; Rodriguez Salvador, A.; Rivarola, M.; Paniego, N.; Nunes-Nesi, A.; Ruiz, O. A.; Sansberro, P. A. Transcript and metabolic adjustments triggered by drought by drought in Ilex paraguariensis leaves. *Planta* **2019**, 250, 445-462. DOI: 10.1007/s00425-019-03178-3
2. Aidoo, M.K.; Quansah, L.; Galkin, E.; Batushansky, A.; Wallach, R.; Moshelion, M.; Bonfil, D.J.; Fait, A. A combination of stomata deregulation and a distinctive modulation of amino acid metabolism are associated with enhanced tolerance of wheat varieties to transient drought. *Metabolomics* **2017**, 13, 138. DOI: 10.1007/s11306-017-1267-y
3. Alves Filho, E.; Braga, L. N.; Silva, L. M. A.; Miranda, F. R.; Silva, E. O.; Canuto, K. M.; Miranda, M. R.; de Brito, E. S.; Zocolo, G. J. Physiological changes for drought resistance in different species of Phyllanthus. *Sci. Rep*. **2018**, 8, 15141. DOI: 10.1038/s41598-018-33496-7
4. Alvarez, S.; Marsh, E.L.; Schroeder, S.G.; Schachtman, D.P. Metabolomic and proteomic changes in the xylem sap of maize under drought. *Plant Cell Environ*. **2008** 31, 325-340. doi: 10.1111/j.1365-3040.2007.01770.x.
5. Aranjuelo, I.; Tcherkez, G.; Jauregui, I.; Gilard, F.; Ancín, M.; Fernandez-San Millan, A.; Larraya, L.; Veramendi, J.; Farran, I. Alteration by thioredoxin over-expression of primary carbon metabolism and its response to elevated $CO_2$ in tobacco (*Nicotiana tabacum* L.). Environmental and *Exp. Bot*. **2015a**, 118, 40-48. DOI: 10.1016/j.envexpbot.2015.05.008
6. Aranjuelo, I.; Erice, G.; Sanz-Sáez, A.; Abadié, C.; Gilard, F.; Gil-Quintana, E.; Avice, J. C.; Staudinger, C.; Wienkoop, S.; Araus, J.L.; Bourguignon, J.; Irigoyen, J. J.; Tcherkez, G. Differential $CO_2$ effect on primary carbon metabolism of flag leaves in durum wheat (*Triticum durum* Desf.). *Plant Cell Environ.* **2015b**, 38, 2780-2794. DOI: 10.1111/pce.12587
7. Arbone, V.; Argamasilla, R.; Gómez-Cadenas, A. Common and divergent physiological, hormonal and metabolic responses of Arabidopsis thaliana and *Thellungiella halophila* to water and salt stress. *J. Plant Physiol*. **2010**, 167, 1342-1350. DOI: 10.1016/j.jplph.2010.05.012
8. Ashrafi, M.; Azimi-moqadam, M. R.; Moradi, P.; MohseniFard, E.; Shekari, F.; Kompany-Zareh, M. Effect of drought stress on metabolite adjustments in drought tolerant and sensitive thyme. *Plant Physiol. Biochem*. **2018**, 132, 391-399. doi.org/10.1016/j.plaphy.2018.09.009
9. Austen, N.; Walker, H. J.; Lake, J. A.; Phoenix, G. K.; Cameron, D. D. The regulation of plant secondary metabolism in response to abiotic stress: Interactions between heat shock and elevated $CO_2$. *Front. Plant Sci*. **2019**, 10, 1463. doi: 10.3389/fpls.2019.01463
10. Barchet, G.L.H.; Dauwe, R.; Guy, R.D.; Schroeder, W.R.; Soolanayakanahally, R.Y.; Campbell, M.M.; Mansfield, S.D. Investigating the drought-stress response of hybrid poplar genotypes by metabolite profiling. *Tree Physiol*. **2013** 34, 1203-1219. DOI: 10.1093/treephys/tpt080
11. Benevenuto, R.F.; Agapito-Tenfen, S.Z.; Vilperte, V.; Wikmark, G.; van Rensburg, P.J.; Nodari, R.O. Molecular responses of genetically modified maize to abiotic stresses as determined through proteomic and metabolomics analyses. *PlosOne* **2017**, 12, e0173069. doi: 10.1371/journal.pone.0173069
12. Berini, J.L.; Brockman, S.A.; Hageman, A.D.; Reich, P.B.; Muthukrishnan, R.; Montgomery, R.A.; Forester, J.D. Combinations of abiotic factors differentially alter production of plant secondary metabolites in five woody plant species in the boreal-temperate transition zone. *Front. Plant Sci*. **2018**, 9, 1257. doi: 10.3389/fpls.2018.01257
13. Cardenas-Manríquez, Vega-Muñoz, I.; Villagómez-Aranda, A. L.; León-Galvan, M. F.; Cruz-Hernandez, A.; Torres-Pacheco, I.; Rangel-Cano, R. M.; Rivera-Bustamante, R. F.; Guevara-Gonzalez, R. G. Proteomic and metabolomic profiles in trangenic tobacco (N. tabacum xanthinc) to CchGLP from Capsicum chinense BG-3821 resistant to biotic and abiotic stresses. *Environ. Exp. Bot*. **2016**, 130, 33-41. doi.org/10.1016/j.envexpbot.2016.05.005
14. Casartelli, A.; Riewe, D.; Hubberten, H.M.; Altmann, T.; Hoefgen, R.; Heuer, S. Exploring traditional aus-type rice for metabolites conferring drought tolerance. *Rice* **2018**, 11, 9. DOI: 10.1186/s12284-017-0189-7
15. Charlton, A.J.; Donarski, J.A.; Harrison, M.; Jones, S.A.; Godward, J.; Oehlschlager, S.; Arques, J.L.; Ambrose, M.; Chinoy, C.; Mullineaux, P.M.; Domoney, C. Response of the pea (*Pisum sativum* L.) leaf metabolome to drought stress assessed by nuclear magnetic resonance spectroscopy. *Metabolomics* **2008**, 4, 312-327. DOI: 10.1007/s11306-008-0128-0

16. Correira, B.; Valledor, L.; Hancock, R. D.; Renaut, J.; Pascual, J.; Soares, A. M. V. M.; Pinto, G. Integrated proteomics and metabolomics to unlock global and clonal responses of Eucalyptus globulus recovery from water deficit. *Metabolomics* **2016**, 12: 141. DOI: 10.1007/s11306-016-1088-4
17. Correia B, Hancock RD, Amaral J.; Gomez-Cadenas A, Valledor L, Pinto G. Combined drought and heat activates protective responses in *Eucalyptus globulus* that are not activated when subjected to drought or heat stress alone. *Front. Plant Sci*. **2018**, 9, 819. doi: 10.3389/fpls.2018.00819
18. Cramer, G. R.; Ergül A.; Grimplet, J.; Tillet, R. L.; Tattersall, E. A. R.; Bohlman, M. C.; Vincent, D.; Sonderegger, J.; Evans, J.; Osborne, C.; Quilici, D.; Schlauch, K. A.; Schooley, D. A.; Cushman, J. C. Water and salinity stress in grapevines: early and late changes in transcript and metabolite profiles. Fucntional and Integrative *Genomics* **2007**, 7, 111-134. DOI: 10.1007/s10142-006-0039-y
19. Creydt, M.; Vulathan-Eckert, J.; Fischer, J. F. M. Effects of elevated $CO_2$ concentration on leaves and berries of black elder (*Sambucus nigra*) using UHPLC-ESI-QTOF-MS/MS and gas exchange measurements. *J. Plant Physiol*. **2019**, 234-235, 71-79. DOI:10.1016/j.jplph.2019.01.005
20. Das, A.; Rushton, P.J.; Rohila, J.S. Metabolomic profiling of soybeans (*Glycine max* L.) reveals the importance of sugar and nitrogen metabolism under drought and heat stress. Plants, **2017**, 6, 21. doi: 10.3390/plants6020021.
21. De Souza, A. P.; Cocuron, J. C.; Garcia, C.; Alonso, A. P.; Buckeridge, M. S. Changes in whole-plant metabolism during the grain-filling stage in sorghum grown under elevated CO2 and drought. *Plant Physiol*. **2015**, 169, 1755-1765. doi.org/10.1104/pp.15.01054
22. Do, P. T.; Degenkolbe, T.; Erban, A.; Heyer, A. G.; Kopka, J.; Köhl, K. I.; Hincha, D. K.; Zuther, E. Dissecting rice polyamine metabolism under controlled long-term drought stress. *PlosOne* **2013** , 8, e60325. doi: 10.1371/journal.pone.0060325
23. Du, H.; Wang, Z.; Yu, W.; Liu, Y.M.; Huang, B. Differential metabolic responses of perennial grass *Cynodon transvaalensis* x *Cynodon dactylon* (C4) and *Poa pratensis* (C3) to heat stress. *Physiol. Plant*. **2011**, 141, 251-264. doi:10.1111/j.1399-3054.2010.01432.x
24. Elber Vital, C.; Giordano, A.; de Almeida Soares E.; Rhys Williams, T. C.; Oliveira Mesquida, R.; Pereira Vidigal, P. M.; de Santana Lopes, A.; Gomes Pacheco, T.; Rogalski, M.; de Oliveira Ramos, H. J.; Loureiro, M. E. An integrative overview of the molecular and physiological responses of sugarcane under drought conditions. *Plant Mol. Biol*. **2017**, 94, 577-594. doi: 10.1007/s11103-017-0611-y
25. Escandón, M.; Meijon, M.; Valledor, L.; Pascual, J.; Pinto, G.; Cañal, M. J. Metabolome integrated analysis of high-temperature response in *Pinus radiata*. *Front. Plant Sci*. **2018**, 9, 485. doi: 10.3389/fpls.2018.00485
26. Evers, D.; Lefevre, I.; Legay, S.; Lamoureux, D.; Hausman, J.F.; Gutierrez Rosales, R.O.; Tincopa Marca, L.R.; Hoffmann, L.; Bonierbale, M.; Schafleitner, R. Identification of drought-responsive compounds in potato through a combined transcriptomic and targeted metabolite approach. *J. Exp. Bot*. **2010**, 61, 2327-2343. doi: 10.1093/jxb/erq060
27. Fernández de Simón, B.; Cadahía, E.; Aranda, I. Metabolic response to elevated CO2 levels in Pinus pinaster Aiton needles in an ontogenic and genotypic-dependent way. Plant Physiology and *Biochemistry* **2018**, 132, 202-212. doi: 10.1016/j.plaphy.2018.09.006
28. Field, K. J. Lake, J. A. Environmental metabolomics links genotype to phenotype and predicts genotype abundance in wild plant populations. *Physiol. plant.* **2011**, 142, 352-360. doi: 10.1111/j.1399-3054.2011.01480.x
29. Fisher, L.H.C.; Han, J.; Corke, F.M.K.; Akinyemi, A.; Didion, T.; Nielsen, K.K.; Doonan, J.H.; Mur, L.A.J.; Bosch, M. Linking dynamic phenotyping with metabolite analysis to study natural variation in drought responses of *Brachypodium distachyon*. *Front. Plant Sci*. **2016**, 7, 1751. doi: 10.3389/fpls.2016.01751
30. Foito, A.; Byrne, S. L.; Shepherd, T.; Stewart, D.; Barth, S. Transcriptional and metabolic profiles of *Lonium perenne* L. genotypes in response to a PEG-induced water stress. *Plant Biotech. J*. **2009**, 7, 719-732. doi: 10.1111/j.1467-7652.2009.00437.x
31. Freitag, S.; Feldmann, J.; Raab, A.; Crittenden, P.D.; Hogan, E.J.; Squier, A.H.; Boyd, K.G.; Thain, S. Metabolite profile shifts in the heathland lichen Cladonia portentosa in response to N deposition reveal novel biomarkers. *Physiol. Plant.* **2012**, 146, 160-172. DOI: 10.1111/j.1399-3054.2012.01593.x
32. Gargallo-Garriga, A.; Jordi Sardans, Míriam Pérez-Trujillo, Albert Rivas-Ubach, Michael Oravec, Otmar Urban, Juergen Kreyling, Anke Jentsch, Carl Beierkuhnlein, Teodor Parella, Josep Peñuelas. Opposite metabolic responses of shoots and roots to drought. *Sci. Rep.* **2014** 4, 6829. DOI: 10.1038/srep06829
33. Gargallo-Garriga, A.; Wright, S. J.; Sardans, J.; Pérez-Terujillo, M.; Ovarec, M.; Vecerova, K.; Urban, O.; Fernandez-Martinez, M.; Parella, T.; Peñuelas, J. Long-term fertilization determines different metabolomic

profiles and responses in saplings of three rainforest tree species with different adult canopy position. *PlosOne* **2017**, 12, e0177030. DOI: 10.1371/journal.pone.0177030

34. Gargallo-Garriga, A.; Preece, C.; Sardans, J.; Oravec, M.; Urban, O.; Peñuelas, J. Root exudate metabolomes change under drought and show limited capacity for recovery. *Sci. Rep*. **2018**, 8, 12696. DOI: 10.1038/s41598-018-30150-0
35. Geng, S.; Misra, B. B.; de Armas, E.; Huhman, D. V.; Alborn, H. T.; Summer, L. W.; Chen, S. Jasmonate-mediated stomatal closure under elevated $CO_2$ revealed by time-resolved metabolomics. *Plant J*. **2016**, 88, 947-962. doi: 10.1111/tpj.13296
36. Georgii, E.; Jin, M.; Zhao, J.; Kanawati, B.; Schmitt-Kopplin, P.; Albert, A.; Winkler, J.B.; Schäffner, A.R. Relationships between drought, heat and air humidity responses revealed by transcriptome-metabolome co-analusis. *BMC Plant Biol*. **2017**, 17, 120. doi: 10.1186/s12870-017-1062-y
37. Glaubitz, U.; Li, X.; Schaedel, S.; Erban, A.; Sulpice, R.; Kopka, J.; Hincha, D. K.; Zulther, E. Integrated analysis of rice transcriptomic and metabolomics responses to elevated night temperatures identifies sensitivity- and tolerant-related profiles. *Plant Cell Environ*. **2017**, 40, 121-137. DOI: 10.1111/pce.12850
38. Griesser, M.; Weingart G.; Schoedl-Hummel, K.; Neumann, N.; Becker, M.; Varmuza, K.; Liebner, F.; Schuhmacher, R.; Forneck, A. Severe drought stress is affecting selected primary metabolites polyphenols, and volatile metabolites in grapevine leaves (*Vitis vinifera* cv. Pinor noir). *Plant Physiol. Biochem*. **2015**, 88, 17-28. DOI: 10.1016/j.plaphy.2015.01.004
39. Heyneke E, Watanable M, Erban A, Duan G, Buchner P, Walther D, Kopka J, Hawkesford MJ, Hosfgen R. Characterization of the wheat leaf metabolome during grain filling and under varied N-supply. *Front. Plant Sci*. **2017**, 8, 2048. doi: 10.3389/fpls.2017.02048
40. Hochberg, U.; Degu, A.; Toubiana, D.; Gendler, T.; Nikoloski, Z.; Rachmilevitch, S.; Fait, A. Metabolite profiling and network analysis reveal coordinated changes in grapevine water stress response. *BMC Plant Biol*. **2013**, 13, 184. doi: 10.1186/1471-2229-13-184.
41. Högy, P.; Keck, M.; Niehaus, K.; Franzaring, J.; Fangmeier, A. Effects of atmospheric $CO_2$ entichment on biomass, yield and low molecular weight metabolites in wheat grain. *J. Cereal Sci*. **2010**, 52, 215-220. DOI: 10.1016/j.jcs.2010.05.009
42. Hu, Y.; Peuke, A.D.; Zhao, X.; Yan, J.; Li, C. Effects of simulated atmospheric nitrogen deposition on foliar chemistry and physiology of hybrid poplar seedlings. *Plant Physiol. Biochem*. **2019**, 143, 94-108. doi.org/10.1016/j.plaphy.2019.08.023
43. Huhn, G.; Schulz, H. Contents of free amino acids in Scots pine needles from field sites with different levels of nitrogen deposition. *New Physiol*. **1996**, 134, 95-101. **doi.org/10.1111/j.1469-8137.1996.tb01149.x**
44. Jia, X.M, Sun, C.; Zuo, Y.; Li, G.; Li, G.; Ren, L.; Chen, G. Integrating transcriptomics and metabolomics to characterize the response of *Astragalus membranaceus* Bge. Var. mongolicus (Bge.) to progressive drought stress. *BMC Genomics* **2016,** 17, 188. doi: 10.1186/s12864-016-2554-0
45. Jia, H.; Wang, L.; Li, J.; Sun, P.; Lu, M.; Hu, J. Comparative metabolomics analysis reveals different metabolomics responses to drought in tolerant and susceptible poplar species. *Physiol. Plant*. **2019** DOI:10.1111/ppl.13036.
46. Jia, H.; Wang, L.; Li, J.; Sun, P.; Lu, M.; Hu, J. Physiological and metabolomics responses of *Salix sinopurpurea* and *Salix suchowensis* to drought stress. *Trees* **2020** DOI:10.1007/s00468-019-01937-z.
47. Ju, Y.L.; Yue, X.F.; Zhao, X.F.; Zhao, H.; Fang, Y.L. Physiological, micro-mosphological and metabolomics analysis of grapevine (*Vitis vinifera* L.) leaf of plants under water stress. *Plant Physiol. Biochem*. **2018** 130, 501-510. DOI: 10.1016/j.plaphy.2018.07.036
48. Kang, Z.; Babar, M.A.; Khan, N.; Guo, J.; Khan, J.; Islam, S.; Shresha, S.; Shahi, D. Comparative metabolomics profiling in the roots and leaves in contrasting genotypes reveals complex mechanisms involved in post-anthesis drought tolerance in wheat. *PlosOne* **2019**, 14, e0213502.
49. Kaplan, F.; Kopka, J.; Haskell, D. W.; Zhao, W.; Schiller, C.; Gatzke, N.; Sung, D. Y.; Guy, C. J. **2004**. Exploring the temperature-stress metabolome of Arabidopsis. *Plant Physiol*. 136, 4159-4168. doi.org/10.1104/pp.104.052142
50. Koscielny, C.B.; Hazebroek, J.; Duncan, W. Phenotypic and metabolomics variation among spring Brassica napus genotypes during heat stress. *Crop Pasture Sci*. **2018**, 10.1071/CP17259.
51. Lang, C. P.; Merkt, N.; Klaiber, I.; Pfannstiel, J.; Zörb, C. Different forms of nitrogen application affect metabolite patternbs in grapevine leaves and the sensory of wine. *Plant Physiol. Biochem*. **2019**, 143, 308-319.

52. Lei, G.; Zhang, H.Y.; Wang, Z.H.; Wei, L.X.; Fu, P.; Song, J.B.; Fu, D.H.; Huang, Y.J.; Liao, J.L. High nighttime temperature induces antioxidant molecule perturbations in heat-sensitive and heat-tolerant coisogenic rice (*Oryza sativa*) strains. *J. Agric. Food Chem.* **2018**, 66, 12131-12140.
53. Lenk I, Fisher LHC, Vickers M, Akinyemi A, Didion T, Swain M, Jensen CS, Mur LAJ, Bosch M. Transcriptional and metabolomics analyses indicate that cell wall properties are associated with drought tolerance in *Brachypodium distachyon*. *Int. J. Mol. Sci*. **2019**, 20, 1758. DOI: 10.3390/ijms20071758
54. Levine, L.H.; H. Kasahara, J. Kopka, A. Erban, I. Fehrl, F. Kaplan, W. Zhao, R.C. Littell, C. Guy, R. Wheeler, J. Sager, A. Mills, and H.G. Levine. Physiologic and metabolic responses of wheat seedlings to elevated and super-elevated carbon dioxide. *Adv. Space R*es. **2008**, 42, 1917-1928.
55. Li, P.; Ainsworth, E.A.; Leakey, A.D.B.; Ulanov, A.; Lozovaya, V.; Ort, D.R.; Bohnert, H.J. *Arabidopsis* transcript and metabolite profiles: ecotype-specific responses to open-air elevated [CO2]. *Plant Cell Environ*. **2008**, 31, 1673-1687.
56. Li, M.; Li, Y.; Zhang, W.; Li, S.; Gao, Y.; Ai, X.; Zhang, D.; Liu, B.; Li, Q. Metabolomics analysis reveals that elevated atmospheric $CO_2$ alleviates drought stress in cucumber seedlings leaves. *Anal. Biochem.* **2018**, 559, 71-85.
57. Liu, T. Y.; Chen, M.X.; Zhang, Y.; Zhu, F.Y.; Liu, Y.G.; Tian, Y.; Fernie, A. R.; Ye, N.; Zhang, J. Comparative metabolite profiling of two switchgrass ecotypes reveals differences in drought stress responses and rhizosheath weight. *Planta* **2019**, 250, 1355-1369.
58. Ma, X.; Xia, H.; Liu, Y.; Wei, H.; Zheng, X.; Song, C.; Chen, L.; Liu, H.; Luo, L. Transcriptomic and metabolomics studies disclose key metabolism pathways contributing to well-maintained photosynthesis under the drought-tolerance in rice. *Front. Plant Sci*. **2016**, 7, 1886. doi: 10.3389/fpls.2016.01886
59. Melandri, G.; Elgawad, H. A.; Riewe, D.; Hageman, J. A.; Asard, H.; Beemster, G. T. S.; Kadam, N.; Jagadish, K.; Altmann, T.; Ruyter-Spira, C.; Bouwmeester, H. Biomarkers for grain yield stability in rice under drought stress. *J. Exp. Bot.* **2020**, 71, 669-683. doi: 10.1093/jxb/erz221
60. Mibei, E.K.; Owino, W.O.; Ambuko, J.; Giovannoni, J.J.; Onyango, A.N. Metabolomic analyses to evaluate the effect of drought stress on selected African Eggplant accessions. *J. Sci. Food Agric*. **2017**, 98, 205-216. **doi.org/10.1002/jsfa.8458**
61. Michailidis, M.; Karagiannis, E.; Tanou, G.; Sarrou, E.; Stavridou, E.; Ganopoulos, I.; Karamanoli, K.; Madesis, P.; Martens, P.; Martens, S.; Malassiotis, A. An integrated metabolomics and gene expression analysis identifies heat and calcium metabolic networks underlying postharvest sweet cherry fruit senescence. *Planta* **2019**, 250, 2009-2022.
62. Michaletti, A.; Naghavi, M.R.; Toorchi, M.; Zolla, L.; Rinalducci, S. Metabolomics and proteomics reveal drought-stress responses of leaf tissues from spring-wheat. Scientific Reports, 2018, 8, 5710.
63. Miyagi, A.; Takahara, K.; Kasajima, I.; Kawai-Yamada, M.; Uchimiya, H. Fate of $^{13}C$ in metabolic pathways and effects of high $CO_2$ on the alteration on mwetabolites in Rumex obtusifolius L. *Metabolomics* **2011**, 7, 524-535. DOI:10.1007/s11306-010-0272-1
64. Moradi, P.; Ford-Lloyd, B.; Pritchard, J. Metabolomic approach reveals the biochemical mechanisms underlying drought stress tolerance in thyme. *Anal. Biochem.* **2017**, 527, 49-62. DOI: 10.1016/j.ab.2017.02.006
65. Moschen, S.; Di Rienzo, J.A.; Higgins, J.; Tohge, T.; Watanable, M.; Gonzalez, S.; Rivarola, M.; García-García, F.; Dopazo, J.; Hopp, H.E.; Hoefgen, R.; Fernie, A.R.; Paniego, N.; Fernández, P.; Heinz, R.A. Integration of transcriptomic and metabolomics data reveals hub transcription factors involved in drought stress response in sunflower (*Helianthus annus* L. ). *Plant Mol. Biol*. **2017**, 94, 549-564. DOI: 10.1007/s11103-017-0625-5
66. Mutwakil, M.Z.; Hajrah, N.H.; Atef, A.; Edris, S.; Sabir, M.J.; Al-Ghamdi, A.K.; Sabir, M.J.S.; Nelson, C.; Makki, R.M.; Ali, H.M.; El-Domyati, F.M.; Al-Hajar, A.S.M.; Gloaguen, Y.; Al-Zahrani, H.S.; Sabir, J.S.M.; Jansen, R.K.; Bahieldin, A.; Hall, N. Transcriptomic and metabolic responses of Calotropis procera to salt and drought stress. *BMC Plant Biol*. **2017**, 17, 231. DOI: 10.1186/s12870-017-1155-7
67. Nakabayashi, R.; Mori, T.; Saito, K. Alteration of flavonoid accumulation under drought stress in Arabidopsis thaliana. *Plant Signal. Beh.* **2014**, 9, e29518. doi: 10.4161/psb.29518
68. Noguchi, K.; Tsunoda, T.; Miyagi, A.; Kawai-Yamada, M.; Sugiura, D.; Miyazawa, S.I.; Tokida, T.; Usui, Y.; Nakamura, H.; Sakai, H.; Hasegawa, T. Effects of elevated atmospheric $CO_2$ on respiratory rates in mature leaves of two rice cultivars grown at a free-air $CO_2$ enrichment site and analyses of the underlying mechanisms. *Plant Cell Environ*. **2018**, 59, 637-649. doi.org/10.1093/pcp/pcy017
69. Obata, T.; Witt, S.; Lisec, J.; Palacios-Rojas, N.; Florez-Sarasa, I.; Yousfi, S.; Araus, J.L.; Cairns, J.E.; Fernie, A.R. Metabolite profiles of maize leaves in drought heat, and combined stress field trials reveal the

relationship between metabolism and grain yield. *Plant Physiol.* **2015**, 169, 2665-2683. DOI: 10.1104/pp.15.01164
70. Paudel, J. R.; Amirizian, A.; Krosse, S.; Giddings, J.; Ismail, S. A. A.; Xia, J.; Gloer, J. B.; van Dam, N. M.; Bede, J. C. Effect of atmopspheric carbon dioxide levels and nitrate fertilization on glucosinolate biosynthesis in mechanically damaged Arabidopsis plants. *BMC Plant Biol*. **2016** , 16, 68.
71. Pavli, O.I.; Vlachos, C.E.; Kalloniati, C.; Flemetakis, E.; Skaracis, G.N. Metabolite profiling reveals the effect of drought on sorghum (*Sorghum bicolor* L. Moench) metabolism. *Plant Omics J*. **2013**, 6, 371-376.
72. Piasecka A, Sawikowska A, Kucynska A, Ogrodowicz P, Mikolajczak K, Krystkowiak K, Gudys K, Guzy-Wróbelska J, Krajewski P, Kachlicki P. Drought-related secondary metabolites of barley (*Hordeum vulgare* L.) leaves and their metabolomics quantitative trait loci. *Plant J*. **2017**, 89, 889-913.
73. Pinasseau, L.; Vallverdú-Queralt, A.; Verbaere, A.; Roques, M.; Meudec, E.; Le Cunff, L.; Péros, J.P.; Ageorges, A.; Sommerer, N.; Boulet, J.C.; Terrier, N.; Cheynier, V. Cultivar diversity of grape skin polyphenol composition and changes in response to drought investigated by LC-MS based metabolomics. *Front. Plant Sci*. **2017**, 8, 1826. doi: 10.3389/fpls.2017.01826
74. Pinheiro, C.; Passarinho, J. A.; Ricardo, C. P. Effect of drought and rewatering on the metabolism of Lupinus albus organs. *J. Plant Physiol*. **2004**, 161, 1203-1210. doi: 10.1016/j.jplph.2004.01.016
75. Pires, M. V.; Pereira Junior, A. A.; Medeiros, D. B.; Daloso, D. M.; Pham, P. A.; Barros, K. A.; Engqvist, M. K.; Florian, A.; Krahnert, I.; Maurino, V. G.; Araújo, W. L.; Fernie, A. R. The influence of alternative pathways of respiration that utilize branched-chain amino acids following water shortage in Arabidopsis. *Plant Cell Environ*. **2016** , 39, 1304-1319. DOI: 10.1111/pce.12682
76. Prins, A.; Mukubi, J. M.; Pellny, T. K.; Verrier, P. J.; Beyene, G.; Lopes, M. S.; Emani, K.; Treumann, A.; Lelarge-Trouverie, C.; Noctor, G.; Kunert, K. J.; Kerchev, P.; Foyer, C.H. Acclimation to high $CO_2$ in maize is related to water status and dependent on leaf rank. *Plant Cell Environ*. **2011**, 34, 314-331.
77. Prinsi, B.; Negri, A.S.; Failla, O.; Scienza, A.; Espen, L. Root proteomic and metabolic analyses reveal specific responses to drought stress in differently tolerant grapevine rootstocks. *BMC Plant Biol*. **2018**, 18, 126.
78. Qi, X.; Xu, W.; Zhang, J.; Guo, R.; Zhao, M.; Hu, L.; Wang, H.; Dong, H.; Li, Y. Physiological characteristics and metabolomics of transgenic wheat containing the maize C4 phosphoenolpyruvate carboxylase (PEPC) gene under high temperature stress. *Protoplasm*a **2017**, 254, 1017-1030. DOI: 10.1007/s00709-016-1010-y
79. Rastogi, S.; Shah, S.; Kumar, R.; Vashisth, D.; Akhtar, M.Q.; Kumar, A.; Dwivedi, U.N.; Shasany, A.K. Ocimum Metabolomics in response to abiotic stresses: cold, flood, drought and salinity. *PlosOne* **2019**, 14, e0210903. Doi:10.1371/journal.pone.0210903
80. Raval, S. S.; Mahatma, M. K.; Chakraborty, K.; Bishi, S. K.; Singh, A. L.; Rathod, K. J.; Jadav, J. K.; Sanghani, J. M.; Mandavia, M. K.; Gajera, H. P.; Golakiya, B. A. Metabolomics of groundnut (*Arachis hypogea* L. ) genotypes under varying temperatures regimes. *Plant Growth Reg*. **2018**, 84, 493-505. DOI: 10.1007/s10725-017-0356-2
81. Ren, S.; Ma, K.; Lu, Z.; Chen, G.; Cui, J.; Tong, P.; Wang, L.; Teng, N.; Jin, B. Transcriptomic and metabolomics analysis of the heat-stress response of Populus tomentosa Carr. *Forests* **2019**,10, 383. **doi.org/10.3390/f10050383**
82. Rivas-Ubach, A.; Sardans, J.; Pérez-Trujillo, M.; Estiarte, M.; Peñuelas. J. Strong relationship between elemental sotichiometry and metabolome in plants. *Proc. Natl Aca. Sci. USA* **2012**, 109: 4181-4186. DOI: 10.1073/pnas.1116092109
83. Rivas-Ubach, A.; Sardans, J.; Gargallo-Garriga, Albert, Parella, T.; Perez-Trujillo, M.; Estiarte, M.; and Penuelas, J. Drought stress enhances folivory by shifting foliar metabolomes in *Quercus ilex* trees. *New Phytol*. **2014**, 202, 874-885.
84. Rivas-Ubach, A.; Barbeta, A.; Sardans, J.; Guenther, A.; Ogaya, R.; Oravec, M.; Urban, O.; Peñuelas, J. Topsoil depth substantially influences the responses to drought of the foliar metabolomics of Mediterranean forests. Persp. Plant Ecol. Evol. Syst. **2016**, 21, 41-54. DOI: 10.1016/j.ppees.2016.06.001
85. Rizhsky, L.; Liang, H.; Shuman, J.; Shulaev, V.; Davletova, S.; Mittler, R. When defense pathways collide. The response of Arabidopsis to a combination of drought and heat stress. *Plant Physiol*. **2004**, 134, 1683-1696.
86. Safronov, O.; Kreuzwieser, J.; Haberer, G.; Alyousif, M.S.; Schulze, W.; Al-Harbi, N.; Arab, L.; Ache, P.; Stempfi, T.; Kruse, J.; Mayer, K.X.; Hedrich, R.; Rennenberg, H.; Salojärvi, J.; Kangasjärvi, J. Detecting early signs of heat and drought stress in Phoenix dactylifera (date palm). *PlosOne* **2017** e0177883.

87. Sánchez, D.H.; Schwabe, F.; Erban, A.; Udvardi, M.K.; Kopka, J. Comparative metabolomics of drought acclimation in model and forage legumes. *Plant Cell Environ*. **2012**, 35, 136-149.
88. Savoi, S.; Wong, D.C.J.; Arapitsas, P.; Miculan, M.; Bucchetti, B.; Peterlunger, E.; Fait, A.; Mattivi, F.; Castellarin, S.D. Transcriptome and metabolite profiling reveals that prolonged drought modulates the phenylpropanoid and terpenoid pathway in white grapes (*Vitis vinifera* L.). *BMC Plant Biol*. 2016,16, 67.
89. Semel, Y.; Schauer, N.; Roessner, U.; Zamir, D.; Fernie, A. R. Metabolite analysis for the comparison of irrigated and non-irrigated field grown tomato of varying genotype. *Metabolomics* **2007**, 3, 289-295.
90. Serrano, N.; Ling, Y.; Bahieldin, A.; Mahfouz, M.M. Thermopriming reprograms metabolic homeostasis to confer heat tolerance. *Sci. Rep*. **2019**, 9, 181.
91. Shahbazy, M.; Moradi, P.; Ertaylan, G.; Zahraei, A.; Kompant-Zarech, M. FTICR mass spectrometry-based multivariate analysis to explore distinctive metabolites and metabolic pathways: A comprehensive bioanalytical strategy toward time-course metabolic profiling of Thymus vulgaris plants responding to drought stress. *Plant Sci*. **2020**, 290, 110257.
92. Shen, J.; Zhang, D.; Zhou, L.; Zhang, X.; Liao, J.; Duan, Y.; Wen, B.; Ma, Y.; Wang, Y.; Fang, W.; Zhu, X. Transcriptomic and metabolomics profiling of Camellia sinensis L. cv. "Suchazao" exposed to temperature stresses reveals modification in protein synthesis and photosynthetic and anthocyanin biosynthetic pathways. *Tree Physiol*. **2019**, 39, 1583-1599.
93. Shi, H.; Jiang C.; Ye, T.; Tan, D.X.; Reiter, R.J.; Zhang, H.; Liu, R.; Chan, Z. Comparative physiological, metabolomics, and transcriptomic analyses reveal mechanisms of improved abiotic stress resistance in bermudagrass [*Cynodon dactylon* (L). Pers] by exogenous melatonin. *J. Exp. Bo*t. **2015**, 66, 681-694. DOI: 10.1093/jxb/eru373
94. Silvente, S.; Sobolev, A. P.; Lara, M. Metabolite adjustments in drought tolerant and sensitive soybean genotypes in response to wáter stress. *PlosOne* **2012**, 7, e38554.
95. Sun, C.B.; Fan, X.W.; Hu H.Y.; Liang, Y.; Huang, Z.B.; Pan, J.L.; Wang, L.; Li, Y.Z. Pivotal metabolic pathways related to water deficit tolerance and growth recovery of whole maize plant. *Plant Omics J*. **2013**, 6, 377-387.
96. Tripathi, P.; Rabara, R.; Reese, R.N.; Miller, M.A.; Rohila, J.S.; Subramanian, S.; Shen, Q. S.; Morandi, D.; Bücking, H.; Shulaev, V.; Rushton, P. J. A toolbox of genes, proteins, metabolites and promoters for improving drought tolerance in soybean includes the metabolite coumestrol and stomatal development genes. *BMC Genomics* **2016**, 17, 102.
97. Tschaplinski, T.J.; Abraham, P.E.; Jawdy, S.S.; Gunter, L.E.; Martin, M.Z.; Engle, N.L.; Yang, X.; Tuskan, G.A. The nature of the progression of drought stress drives differential metabolomic responses in *Populus deltoids*. *An. Bot*. **2019**,124, 617-626.
98. Ullah, N.; Yüce, M.; Gökçe, Ö.; Budak, H. Comparative metabolite profiling of drought stress in roots and leaves of seven Triticeae species. *BMC Genomics* **2017**, 18, 969.
99. Vazquez-Robinet, C.; Mane, S.P.; Ulanov, A.V.; Watkinson, J.I.; Stromberg, V.K.; De Koeyer, D.; Schafleitner, R.; Willmot, D.B.; Bonierbale, M.; Bohnert, H.J.; Grene, R. Physiological and molecular adaptations to drought in Andean potato genotypes. *J. Exp. Bot*. **2008**, 59, 2109-2123.
100. Wang, L.; Ma, H.; Song, L.; Shu, Y.; Gu, W. Comparative proteomics analysis reveals the mechanism of pre-harvest seed deterioration of soybean under high temperature and humidity stress. *J. Proteomics* **2012**, 75, 2109-2127.
101. Wang, X.; Hou, L.; Lu, Y.; Wu, B.; Gong, X.; Liu M.; Wang, J.; Sun, Q.; Vierling, E.; Xu, S. Metabolic adaptation of wheat grain contributes to a stable filling rate under heat stress. J. Exp. Bot. **2018**, 69, 5531-5545. doi: 10.1093/jxb/ery303
102. Wang, X.; Guo, R.; Li, M.; Liu, Y.; Zhao, M.; Fu, H.; Liu, X.; Wang, S.; Shi, L. Metabolomics reveals the drought-tolerance nechanism in wild soybean (Glycine soja). *Acta Physiol. Plant*. **2019**, 41, 161.
103. Waren, C.R.; Aranda, I.; Cano, F.J. Metabolomics demonstrates divergent responses of two Eucalyptus species to water stress. *Metabolomics* **2012**, 8, 186-200.
104. Wedeking, R.; Maucourt, M.; Deborde, C.; Moing, A.; Gibon, Y.; Goldbach, H.E.; Wimmer, M.A. $^1$H-NMR metabolomics profiling reveals a distinct metabolic recovery response in shoots and roots of temporarily drought-stressed sugar beets. *PlosOne* **2018**, 13, e0196102.
105. Wedow, J.M.; Yendrek, C.R.; Mello, T.R:, Creste, S.; Martinez, C.A.; Ainsworth, E.A. Metabolite and transcript profiling of Guinea grass (*Panicum maximum* Jacq) response to elevated [$CO_2$] and temperature. *Metabolomics* **2019**, 15, 51.

106. Witt, S.; Galicia, L.; Lisec, J.; Cairns, J.; TIessen, A.; Araus, J.L.; Palacios-Rojas, N.; Fernie, A.R. Metabolomic and phenotypic responses of greenhouse-grown maize hybrids to experimentally controlled drought stress. *Mol. Plant* **2012**, 5, 401-417. DOI: 10.1093/mp/ssr102
107. Xiong Q, Cao C, Shen T, Zhong L, He H, Chen X. Comprehensive metabolomics and proteomic analysis in biochemical metabolic pathways of rice spikes under drought and submergence stress. *Proteins Proteomics* **2019**, 1867, 237-247.
108. Yadav AK, Caroll AJ, Estavillo GM, Rebetzke GJ, Pogson BJ. Wheat drought tolerance in the field is predicted by amino acid responses to glasshouse-imposed drought. *J. Exp. Bot.* **2019**, 70, 4931-4947.
109. Ye, T.; Shi H, Wang Y, Yang F, Chan Z. Contrasting proteomic and metabolomics responses of bermudagrass to drought and salt stresses. Front. Plant Sci. **2016**, 7, 1694.
110. Yobi, A.; Wone, B.W.M.; Xu, W.; Alexander, D.C.; Guo, L.; Ryals, J.A.; Oliver, M.J.; Cushman, J.C. Metabolomic profiling in Selaginella lepidophylla at various hydration states provides new insights into the mechanistic basis of desiccation tolerance. *Mol. Plant* **2013**, 6, 369-385.
111. You, J.; Zhang, Y.; Liu, A.; Li, D.; Wang, X.; Dossa, K.; Zhou, R.; Yu, J.; Zhang, Y.; Wang L.; Zhang, X. Transcriptomic and metabolomics profiling of drought-tolerant and susceptible sesame genotypes in response to drought stress. *BMC Plant Biol*. **2019**, 19, 267.
112. Zhang, J. Y.; Cruz de Carvalho, M. H.; Torres-Jerez, I.; Kang, Y.; Allen, S.N.; Huhman, D.V.; Tang, Y.; Murray, J.; Summer, L. W.; Udardi, M. K. **2014.** Global reprogramming of transcription and metabolism in *Medicago truncatula* during progressive drought and after rewatering. *Plant Cell Environ*. 37, 2553-2576. doi: 10.1111/pce.12328
113. Zhang, J.; Chen, G.; Zhao, P.; Zhou, Q.; Zhao, X. The abundance of certain metabolites responds to drought stress in the highly drought tolerant plant Caragana korshinskii. *Acta Physiol. Plant.* **2017**, 39, 116. DOI: 10.1007/s11738-017-2412-y
114. Zhuang, L.; Yang, Z.; Fan, N.; Yu, J.; Huang, B. Metabolomic changes associated with elevated $CO_2$-regulation of salt tolerance in Kentucky bluegrass. Environ. Exp. Bot. **2019**, 165, 129-138. DOI:10.1016/j.envexpbot.2019.05.023
